# Control of morphogenesis during the *Staphylococcus aureus* cell cycle

**DOI:** 10.1126/sciadv.adr5011

**Published:** 2025-04-11

**Authors:** Mariana Tinajero-Trejo, Matthew Aindow, Laia Pasquina-Lemonche, Lucia Lafage, Abimbola Feyisara Adedeji-Olulana, Joshua A. F. Sutton, Katarzyna Wacnik, Yaosheng Jia, Bohdan Bilyk, Wenqi Yu, Jamie K. Hobbs, Simon J. Foster

**Affiliations:** ^1^ School of Biosciences, University of Sheffield, Sheffield, UK.; ^2^ The Florey Institute for Host-Pathogen Interactions, University of Sheffield, Sheffield, UK.; ^3^ Department of Physics and Astronomy, University of Sheffield, Sheffield, UK.; ^4^ Department of Molecular Biosciences, University of South Florida, Tampa, FL, USA.

## Abstract

Bacterial cell division is a complex, multistage process requiring septum development while maintaining cell wall integrity. A dynamic, macromolecular protein complex, the divisome, tightly controls morphogenesis both spatially and temporally, but the mechanisms that tune septal progression are largely unknown. By studying conditional mutants of genes encoding DivIB, DivIC, and FtsL, an essential trimeric complex central to cell division in bacteria, we demonstrate that FtsL and DivIB play independent, hierarchical roles coordinating peptidoglycan synthesis across specific septal developmental checkpoints. They are required for the localization of downstream divisome components and the redistribution of peptidoglycan synthesis from the cell periphery to the septum. This is achieved by positive regulation of septum production and negative regulation of peripheral cell wall synthesis. Our analysis has led to a model for the coordination of cell division in *Staphylococcus aureus*, forming a framework for understanding how protein localization and function are integrated with cell wall structural dynamics across the bacteria.

## Teaser

*Staphylococcus aureus* tightly controls division proteins to maintain cell morphology during the cell cycle.

## INTRODUCTION

Cell division requires the formation of a septum that allows scission resulting in two daughter cells. This process is tightly orchestrated by a complex group of proteins required for the septal synthesis of cell wall components while maintaining cell envelope integrity. There are considerable advances in understanding the roles of the protein components involved in cell division, collectively called “the divisome” (*1*–*3*). However, the spatiotemporal control mechanisms that tune septal progression are largely unknown. Across the bacteria, there is a high degree of conservation of the divisome proteins, indicating fundamental similarities in the mechanisms of cell division and a common evolutionary origin (*4*–*6*). Our understanding of cell division mechanisms is derived from studies on a range of “model” and other organisms, each providing insight into this fundamental process of life (*3*).

Morphogenesis during division is dictated by the cell wall, whereby its essential function of maintaining integrity in the face of internal turgor pressure is juxtaposed with dynamics to allow septum formation (*7*–*9*). The Gram-positive, spheroid bacterium *Staphylococcus aureus* has a three-layered cell envelope: the cytoplasmic membrane, the exoplasm, and the 20- to 40-nm-thick cell wall (*10*, *11*). The major structural component of the cell wall for *S. aureus*, and most other bacteria, is the polymer peptidoglycan (PG), which is formed of glycan chains highly cross-linked via peptide side chains. The cell envelope also contains phosphate-rich glycopolymers either in the exoplasm bound to the membrane [lipoteichoic acid (LTA)] or covalently bound to PG [wall teichoic acid (WTA)] (*12*–*14*).

Cell division is a complex series of steps, each delineated by cell wall morphological checkpoints. Initiation of division is marked by the polymerization of the tubulin homolog FtsZ into protofilaments forming a ring structure at mid-cell (the Z-ring), catalyzing the translocation and assembly of early division proteins (*15*–*17*), which then trigger the recruitment of the late division proteins required for septum formation. In *S. aureus*, the first stage of septum formation is characterized by the synthesis of a thin band of PG called the piecrust, which forms the foundation of the septum (*18*). PG synthesis then switches to septal plate formation. This has two characteristic PG architectures, with, at its core, highly ordered concentric rings surrounded by a fine mesh of randomly oriented glycan strands (*18*–*20*). The formation of these structures ultimately leads to closure of the septal annulus, maturation of the septum, and lastly hydrolysis-mediated cell scission resulting in two daughter cells (*21*). Scission reveals the PG ring architecture at the center of the developing septum on the external face of the daughter cells, marking the most recent plane of division (*19*, *22*). Thus, PG dynamics determine morphogenesis during division. The final stages of PG assembly occur in the exoplasm and are driven by transglycosylation and transpeptidation reactions that catalyze the incorporation of the PG building blocks (muropeptides) from lipid II into the existing macromolecular structure. Lipid II is initially flipped across the cell membrane via the highly conserved MurJ (*23*). Transglycosylases (TGs) link the sugars together to form glycan chains, and transpeptidases (TPs) lastly cross-link the nascent material within the macromolecular PG structure to allow growth and division (*2*). *S. aureus* has four PG assembly TPs, which are the penicillin-binding proteins (PBPs) [PBP1 to PBP4; (*2*)]. PBP1 and PBP3 are class B PBPs with TP activity only, PBP2 is a class A PBP with TP and TG activities, and PBP4 is a class C PBP with d,d-carboxypeptidase and TP activity (*2*, *20*). PBP1 and PBP3 require partner TGs (FtsW and RodA, respectively) to allow PG assembly. Cell division and the subtle elongation resulting in the prolate spheroid cell morphology require PBP1/FtsW and PBP3/RodA, respectively (*24*). The TP activity of PBP4 produces the high level of cross-linking in mature PG of *S. aureus*, providing cell envelope stiffness (*25*, *26*). Only PBP1 and PBP2 are essential and can support cell division in the absence of the dispensable PBP3 and PBP4 (*2*, *20*). We first hypothesized, and have very recently demonstrated, that PBP1 activity is responsible for the septal PG ring architecture and that PBP2 can cover all other TP reactions required for morphogenesis (*11*, *27*). Cells lacking PBP1 activity can make a piecrust (*11*, *27*), and so we suggest that this initial defining feature of the septum is created by PBP2 activity.

Growth and division require both cell wall synthesis and hydrolysis to allow areal expansion and separation. Cell wall homeostasis is essential as inhibition of PBP TP activity by the action of β-lactam antibiotics results in cell death due to continued PG hydrolysis in the absence of synthesis (*28*). PG synthesis with reduced hydrolysis is also lethal, but loss of both PG synthesis and hydrolysis results in stasis (*28*). This reveals the need for a precise coordination of activities to balance cell wall homeostasis with morphogenesis. This must also be dynamic as, during division, there are specific structures formed with a high degree of coordination both spatially and temporally. This raises the question as to how cell division is regulated through the series of morphological and structural checkpoints from piecrust formation to septal synthesis, annulus closure, septal maturation, and eventual scission. Although there are morphological differences across the bacteria, they have the same basic coordination requirements, suggesting that regulatory processes may well be conserved at the divisome level.

Conservation of the essential, late cell division, membrane-associated proteins DivIB, DivIC, and FtsL, as well as their capacity to form a trimeric complex in Gram-positive and their orthologs (FtsQ, FtsB, and FtsL, respectively) in Gram-negative bacteria alludes to a common functional importance (*4*–*6*). We have previously established that, in *S. aureus*, DivIB binds PG and is required for septal closure and control of cell size (*29*), whereas DivIC is a WTA-dependent cell wall–binding protein, mediating cell wall dynamics by facilitating the translocation of PBP2 and PBP1 to the division site for septum formation and peripheral cell wall thickness (*30*). These data strongly suggest regulatory functions for the DivIB/DivIC/FtsL complex in morphogenesis; however, the role of *S. aureus* FtsL has not been determined, making it difficult to understand the individual and coordinated roles of these proteins. Here, we reveal that FtsL is required to pass an early checkpoint in septum formation, facilitating the translocation of DivIC and PBP2 to the division site. FtsL and DivIB, while positively regulating septal progression, also coordinate peripheral cell wall synthesis to optimize morphogenesis. On the basis of this information, coupled with functional analysis of the other essential PG assembly components, we have developed a model of the spatial and temporal control of cell division in *S. aureus*, providing a template for other organisms.

## RESULTS

### *S. aureus* requires FtsL for survival, cell morphology, and septum development

**Fig. 1.**
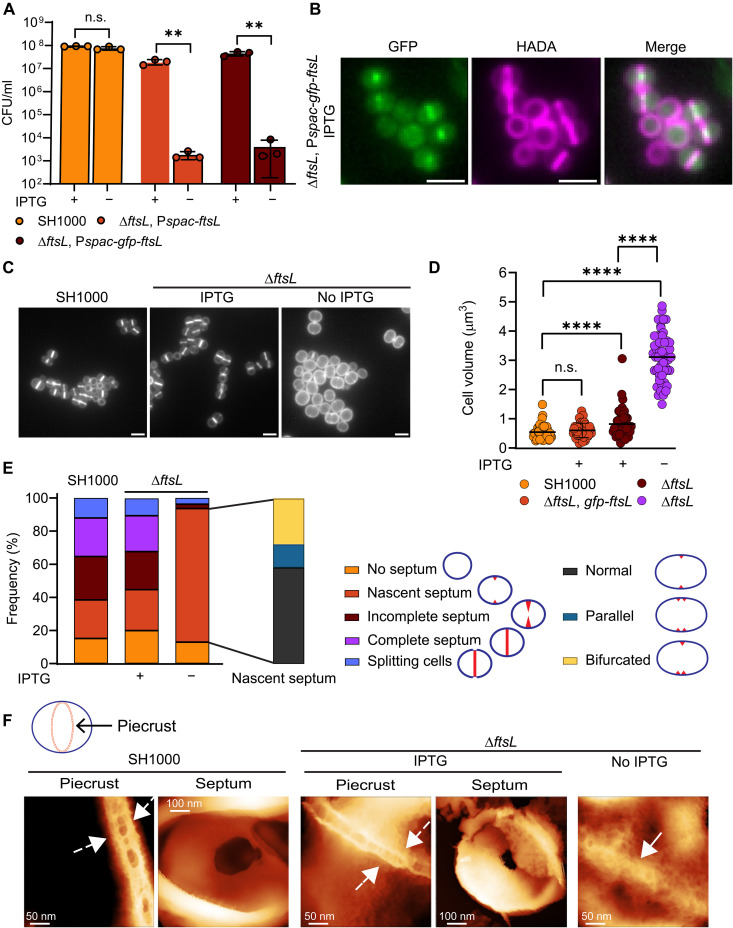
FtsL is required for cell survival, size regulation, and septum development. (**A**) Plating efficiency of SH1000 and Δ*ftsL*, P*spac-ftsL* (SJF5665) and Δ*ftsL*, P*spac-gfp-ftsL* (SJF5666) grown for 2 hours with IPTG followed by overnight growth in solid medium with and without the inducer. Data are the mean and SD of three independent experiments. *P* values were determined by two-tailed unpaired *t* test (from left to right, **P = 0.0027 and 0.0012). n.s., not significant. (**B**) Fluorescence microscopy images of Δ*ftsL*, P*spac-gfp-ftsL* (SJF5666) grown for 1 hour with IPTG. Cells labeled with HADA for 30 min show cell morphology. (**C**) Fluorescence microscopy images of SH1000 and Δ*ftsL* (SJF5665) cells grown for 1 h with or without IPTG. PG was labeled by 30-min incubation with HADA. Images are average *z*-stack intensity projections. Scale bars, 2 μm. Images are representative of two independent experiments. (**D**) Cell volumes of SH1000 and Δ*ftsL* cells determined from images of cells stained with NHS-ester 555 (fig. S1E). Each circle indicates a single cell. *n* = 50 cells per sample. *P* values were determined by Mann-Whitney *U* tests (****P < 0.0001). (**E**) Classification of cell stages of division based on 30-min HADA labeling [as shown in (C)] (left) and septal defects classification (right). *n* = 300 cells per sample. (**F**) AFM descriptive comparison between images of SH1000 and Δ*ftsL* (SJF5665) sacculi of cells grown for 1 hour with or without IPTG. Images show the structural features of piecrust in SH1000, cells expressing FtsL (dashed arrows), and in FtsL-depleted cells (solid arrow). Incomplete septa from the parental and Δ*ftsL* (SJF5665) strains grown with IPTG are shown (cells with incomplete septum were absent in the absence of the inducer).

A transposon-mediated differential hybridization screen identified *ftsL* (SAOUHSC_ 01144), as putatively essential in *S. aureus* (*31*). We constructed an *ftsL* conditional lethal strain in the methicillin-sensitive *S. aureus* (MSSA) strain SH1000 by inserting an additional ectopic copy of *ftsL* under control of the isopropyl-β-D-thiogalactopyranoside (IPTG)–inducible promoter (P*spac*) at the lipase locus (*geh::*P*spac-ftsL*). Because the 3′ sequence of the *ftsL gene* and the *pbp1* promoter overlap, we replaced only the initial part of *ftsL* [222 base pairs (bp)] with a tetracycline resistance cassette leaving the sequence for the normal expression of *pbp1* (fig. S1A) (*27*) (this conditional mutant is henceforth referred to as Δ*ftsL*). In the presence of IPTG, wild type and Δ*ftsL* exhibited comparable plating efficiency on agar, but the absence of the inducer severely compromised the survival of Δ*ftsL* (~0.1% growth compared to the control), indicating *ftsL* essentiality (Fig. 1A). Growth stopped and viability decreased after 1-hour incubation of Δ*ftsL* cells without IPTG, whereas cultures of the isogenic strain (SH1000) and Δ*ftsL* plus inducer grew at a comparable rate (fig. S1B). Loss of FtsL in cells grown for 1 hour in the absence of IPTG was demonstrated by Western blot (fig. S1, C and D). Expression of an FtsL fluorescent fusion (Δ*ftsL geh::*P*spac-gfp-ftsL*) complemented the loss of native FtsL in the presence of IPTG (Fig. 1A and fig. S1B), and fluorescence microscopy demonstrated a septal localization for FtsL in *S. aureus* (Fig. 1B).

Morphological analysis of FtsL-depleted cells revealed a distinctive phenotype (Fig. 1C) of enlarged (Fig. 1D and fig. S1E) and apparent lozenge-like cells. A slight increase in the size of Δ*ftsL* cells in the presence of an inducer (+IPTG) is likely due to differences in the level of expression of the P*spac-ftsL* compared to the parental strain (~50% less expression; fig. S1, C and D). We next tested the ability of FtsL-depleted cells to go through the stages of the cell cycle by analyzing microscopy images of SH1000 and Δ*ftsL* cells grown for 1 hour in the presence or absence of IPTG and labeled with the fluorescent d-amino acid 7-hydroxycoumarin-3-carboxylic acid-amino-D-alanine (HADA) (*32*) to follow PG synthesis. Populations of parental and Δ*ftsL* cells grown with the inducer showed a comparable proportion of cells in the different stages of cell division (Fig. 1, C and E). On the other hand, most of the FtsL-depleted cells (~81%) showed only a small accumulation of PG at the mid-cell and remained at the nascent septum stage, suggesting inability to develop a septal plate (Fig. 1, C and E). In ~55% of these cells, we observed a single septal initiation (as would be expected as a transient stage for SH1000); however, the rest of the population showed two or more initiation sites (Fig. 1E).

Cells lacking DivIB are blocked in the completion but not the initiation of septum formation (*29*). To rule out the possibility that FtsL-depleted cells were not blocked at septum initiation but instead required more time for septal development, we performed a pulse-chase experiment sequentially labeling with two different fluorescent d-amino acid derivatives (FDAs) (*20*, *32*, *33*). After 1-hour incubation with and without IPTG, we added HADA to Δ*ftsL* cells for 5 min to label initiation of PG synthesis at mid-cell followed by 15-min incubation in FDA-free medium. Last, azido-D-alanyl-D-alanine (ADA-DA) was added for a further 5 min to observe any synthesis at the site of septation. The parental strain, an IPTG-dependent *divIB* conditional lethal (Δ*divIB*) (*29*), and the Δ*ftsL* strain grown in the presence of the inducer (IPTG) progressed through the cell cycle as expected (fig. S2A). In the absence of the inducer, pulse-chase fluorescence imaging of PG synthesis using HADA and ADA-DA revealed that FtsL-depleted cells cannot form a septum, whereas DivIB-depleted cells develop a septal plate but are unable to close the annulus, as previously observed (*29*) (fig. S2A).

By transmission electron microscopy (TEM), we observed a clear invagination of the membrane and an accumulation of PG at the cell division site in cells depleted of FtsL, confirming septal initiation but not completion (fig. S1, F and G). The first PG structure formed at the initiation of septation is the piecrust, on which the septal plate is subsequently assembled (*18*, *19*). Analysis of sacculi derived from FtsL-depleted cells, using high-resolution atomic force microscopy (AFM) in liquid, revealed a band of PG corresponding to the piecrust (Fig. 1F and fig. S1H). However, the structure is wider, much more disordered, and appears more open when compared to the piecrust of the parental strain or the Δ*ftsL* plus IPTG controls (Fig. 1F and fig. S1H).

FtsW is an essential, core divisome component required, along with PBP1, for septum formation (*24*). Depletion of FtsW in a conditional lethal strain constructed in the MRSA background COL led to enlarged, elongated cells with unfinished septa in the absence of the inducer IPTG (*24*), reminiscent of our Δ*ftsL* strain. However, there are important differences between MRSA and MSSA backgrounds where the presence of *mecA* (encoding PBP2A) has a profound effect on cellular physiology (*34*). Also, COL harbors a mutation in *rpoB* that confers high-level antibiotic resistance (*34*) and leads to a significant decrease in cell volume in the absence of antibiotics compared to SH1000 (*27*, *35*). For *S. aureus*, therefore, morphogenesis studies in both MSSA and MRSA strains are important. Thus, we constructed and characterized an *ftsW* conditional lethal strain in the MSSA strain SH1000 by introducing an extra copy of the gene into the *geh* locus under control of P*spac* (*geh::*P*spac*), followed by the exchange of the native gene with a tetracycline resistance cassette (Δ*ftsW*) (fig. S3A). FtsW is essential for growth and survival, whereby Δ*ftsW* lost viability upon FtsW depletion (fig. S3, B to D) after 1-hour incubation in the absence of IPTG (fig. S3B). After this time, we observed the presence of several septum initiation sites (figs. S3, E and F, and S4A) and a significant increase in size (fig. S3G). However, the septal structures in the absence of FtsW (figs. S1G and S4, A and B) are more developed than those observed in cells lacking FtsL (Fig. 1C and fig. S1, F and G). By both fluorescence microscopy and TEM, we mainly observed partially formed septa with a distinct V-shape but there appear to be an inability to close the septal annulus, indicating a further septal progression than FtsL-depleted cells (figs. S1G and S4B). High-resolution AFM images of septa from FtsW-depleted sacculi revealed more complex structures. For example, monoseptal cells showed a piecrust comparable to those observed in the parental strain, and cells show dual septa with parallel irregular underdeveloped piecrusts of variable thicknesses (fig. S4C). This indicates that depletion of FtsW leads to altered and irregular septation initiation in *S. aureus*. Thus, loss of FtsL or FtsW did not lead to an identical phenotype, indicating that they are required for different stages during division.

### FtsL is essential for the localization of DivIC and PBP2 to mid-cell during cell division

**Fig. 2.**
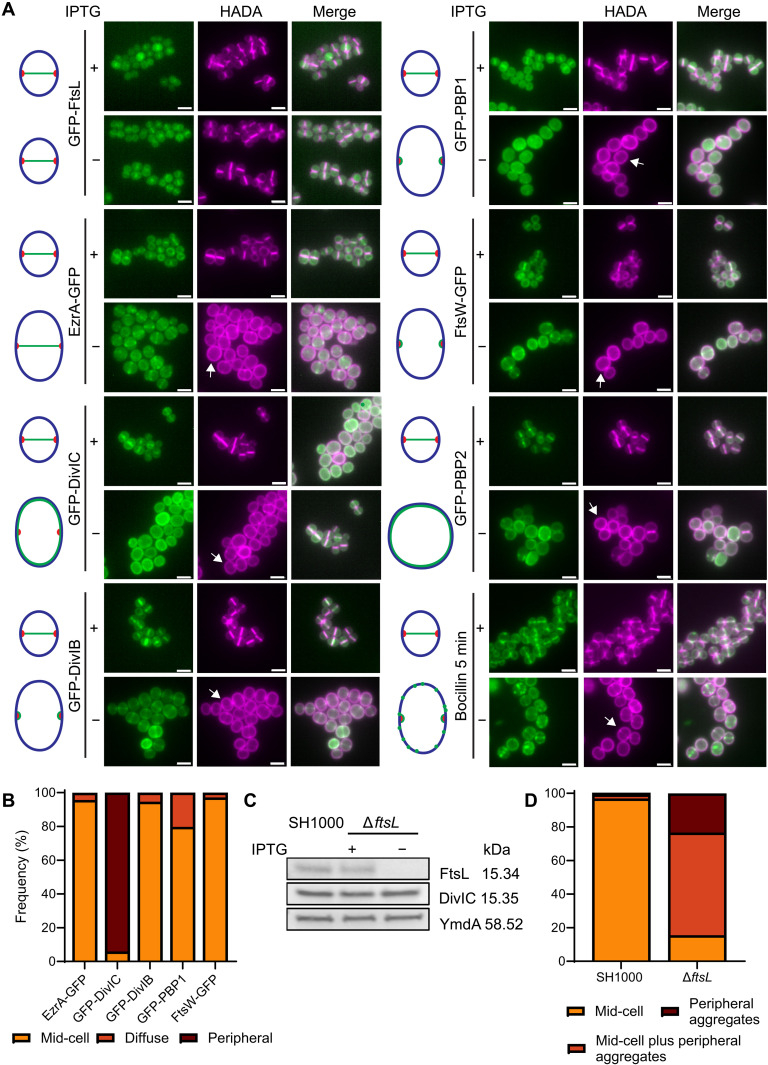
FtsL is essential for the localization of DivIC and PBP2 to mid-cell during cell division. (**A**) Fluorescence microscopy images of Δ*ftsL* cells expressing fluorescent fusions of *ftsL* (GFP-FtsL, SJF5936), *ezrA* (EzrA-GFP, SJF5696), *divIC* (DivIC-GFP, SJF5693), *divIB* (GFP-DivIB, SJF5694), *pbp1* (GFP-PBP1, SJF5932), *ftsW* (FtsW-GFP, SJF5769), and *pbp2* (GFP-PBP2, SJF5695) grown for 1 hour in the presence (+) or absence (−) of IPTG. PG was labeled by 30-min incubation with HADA. Δ*ftsL* was labeled with Bocillin for 5 min with or without IPTG (right, bottom panel). Images are average *z*-stack intensity projections. Scale bars, 2 μm. Images are representative of two independent experiments. Diagrams depict the most frequent patterns of the fluorescent fusion localization and Bocillin (mid-cell or peripheral) (green), the overall cell morphology (blue), and the mid-cell position (red). White arrows show peripheral HADA labeling. (**B**) Frequency of fluorescent fusion localization in Δ*ftsL* cells in the absence of IPTG [based on images in (A)]. Only cells showing incorporation of HADA at mid-cell were considered. *n* > 100 cells per sample. Mid-cell localization of the fluorescent fusions in the parental strain is shown in fig. S5 (A and B). (**C**) Western blot of whole-cell lysates of SH1000 and Δ*ftsL* (SJF5665) grown for 1 hour in the presence or absence of IPTG. Anti-FtsL and anti-DivIC antibodies were used for detection. Detection of YmdA with anti-YmdA antibodies is shown as loading control. Results are representative of three biological repeats (fig. S2A shows signal quantification). (**D**) Frequency of localization of fluorescent Bocillin in Δ*ftsL* cells (SJF5665) [based on images in (A), right-bottom panel]. Frequency of Bocillin localization in the parental strain is shown for comparison. Only cells showing incorporation of HADA at mid-cell were considered. *n* > 150 cells per sample.

Loss of FtsL leads to cells arrested at the piecrust stage of septal development (Fig. 1E and fig. S1G), suggesting that this protein facilitates progression to the next stage of septal plate formation. Thus, FtsL may act as a checkpoint regulator controlling the essential transition to the synthesis of the large amounts of PG required for the development of the septal plate. To explore this, we carried out a combination of fluorescent fusion localization (Fig. 2, A and B) and protein level analysis (Fig. 2C). We expressed early and late division protein fusions in the Δ*ftsL* strain grown without IPTG and added HADA to identify cells with the typical nascent septum structure of FtsL-depleted cells by fluorescence microscopy. Cells grown in the absence of IPTG but expressing a plasmid-based *gfp-ftsL* fusion under control of a cadmium-inducible promoter (P*cad*; see Materials and Methods) showed a phenotype comparable to that of cells grown in the presence of the inducer demonstrating complementation (Fig. 2A). Depletion of FtsL did not affect the mid-cell localization of an ectopically expressed EzrA–green fluorescent protein (GFP) fusion, used as a proxy for the early division protein FtsZ (*15*, *16*). Conversely, expression of a functional *gfp-divIC* fusion in a low copy plasmid under control of the constitutive penicillinase promoter [pLOW-P*pcn-gfp-divIC*; (*30*)] remained peripheral in most of the analyzed cells (~95%) grown in the absence of IPTG but had expected septal localization in cultures containing the inducer and in the parental strain (Fig. 2, A and B, and fig. S5, A and B). According to previous reports, DivIB, DivIC, and FtsL form a complex and are required for each other’s stability (*6*, *36*); however, in the absence of FtsL, DivIC remained stable (Fig. 2C and fig. S2B). Functional, fluorescent fusions of DivIB (fig. S6A) and FtsW (fig. S7A) [pLOW-P*pcn-gfp-divIB* and pLOW-P*pcn-ftsW-gfp*, respectively; (*30*)] had overall mid-cell localization in the absence of the inducer in Δ*ftsL* cells that show some PG incorporation at mid-cell (piecrust) (Fig. 2, A and B). The GFP-PBP1 fusion (pLOW-P*cad-gfp-pbp1*; see Materials and Methods) also localized at mid-cell in ~80% of FtsL-depleted cells with PG septal incorporation, whereas the others (~20%) showed a diffuse signal. Mid-cell localization of these fusions in the parental strain was also observed in cells with septal PG synthesis (Fig. 2, A and B, and fig. S5, A and B).The localization of GFP-PBP2 [P*pcn-gfp-pbp2*; (*30*, *37*)] in the IPTG added Δ*ftsL* and the parental strain was septal; however, in the absence of the inducer, the expression of GFP-PBP2 to the levels that allowed us to see fluorescence led to a morphological alteration characterized by round cells with no identifiable piecrust structures and clear peripheral PBP2 localization (no HADA incorporation at mid-cell) (Fig. 2A). To overcome this, Δ*ftsL* cells with or without IPTG were labeled with fluorescent penicillin (Bocillin) for 5 min to bind to the native PBPs. PBP2 is responsible for the large majority of Bocillin binding and accounts for most of the PG synthesis in *S. aureus* (fig. S5C) (*38*). Approximately 77% of FtsL-depleted cells bound Bocillin at mid-cell and most of them (~85%) also showed distinct peripheral fluorescent aggregates that we did not observe in the control cultures (Fig. 2, A and D, and fig. S5C). We have previously reported the PBP2 dependence on DivIC to localize to mid-cell (*30*). Thus, we propose that FtsL facilitates the localization of DivIC, and hence PBP2, to mid-cell for normal septum development. Peripheral HADA labeling in cells lacking FtsL suggests delocalized activity of PBP2 around the cell as septum formation is inhibited (Fig. 2A). More than 80% of cells showed septal localization of the functional GFP-FtsL fusion (Fig. 2A) when we expressed it in conditional lethal strains depleted of PBP2, FtsW, DivIB, or DivIC (fig. S8, A to D). This indicates that FtsL participates at an early stage in the control of septum development.

Unlike that shown in Δ*ftsL* cells, in DivIB-lacking cells, expression of the GFP-PBP2 fusion showed mid-cell localization (fig. S6, A and B). We also observed septal localization of the GFP-PBP1 fusion in only ~55% of DivIB-depleted cells (fig. S6, A and B), whereas the parental strain showed GFP-PBP1 at the septum in ~96% of cells (fig. S5, A and B). In comparison, GFP-PBP1 fusion showed septal localization in ~80% of cells lacking FtsW whereas EzrA, DivIC, FtsL, and PBP2 fluorescent fusion localization was similar in the Δ*ftsW* and the parental strain (figs. S5, A and B, and S7, A and B). Thus, DivIB seems to be involved in the localization of PBP1 to mid-cell during septal development.

### The C-terminal domain of FtsL is essential for function

**Fig. 3.**
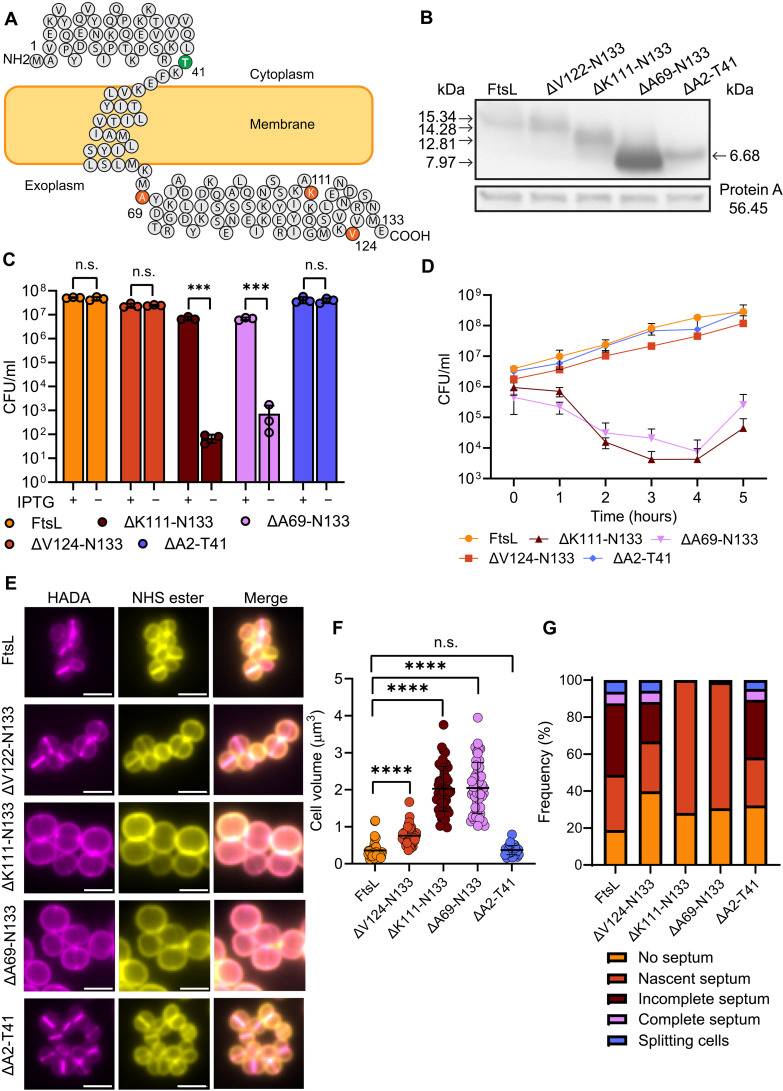
The C-terminal domain of FtsL is essential for function. (**A**) Topological model of FtsL. Amino acids marking the end of each deletion construct are shown for the exoplasmic (red) and the cytoplasmic (green) domains. (**B**) FtsL (SJF5781) or truncated versions of the protein from the C-terminal [ΔV122-N133 (SJF5782), ΔK111-N133 (SJF5783), or ΔA69-N133 (SJF5785)] or the N-terminal domain (ΔA2-T41, SJF5803) detected by Western blot. Whole-cell lysates of cultures grown for 1 hour without IPTG were detected using anti-FtsL antibodies. Anti–protein A antibodies were used as a control. (**C**) Viability test of Δ*ftsL* cells expressing FtsL or the truncated versions of the protein. Cells were grown for 1 hour in the presence and absence of IPTG followed by growth in solid medium with IPTG. Data are the mean and SD of three independent experiments. *P* values were determined by two-tailed unpaired *t* test (from left to right, ***P = 0.0003 and 0.0001). (**D**) Survival of Δ*ftsL* cells expressing FtsL or the truncated versions of the protein grown in the absence of IPTG followed by growth on solid medium with IPTG. Means and SDs of three independent experiments are shown. (**E**) Fluorescence microscopy images of Δ*ftsL* cells expressing FtsL or truncated versions of the protein after growing for 1 hour in the absence of IPTG. Cells labeled with HADA for 30 min and stained with NHS-ester 555 show cell morphology. (**F**) Volume determined from cells stained with NHS-ester 555 [based on images in (E)]. Each circle indicates a single cell. *n* = 50 cells per sample. *P* values were determined by Mann-Whitney *U* tests (****P < 0.0001). (**G**) Classification of cell stages of division based on HADA labeling [based on images in (E)]. *n* > 200 cells per sample.

The predicted topology of FtsL (133 amino acids) in *S. aureus* consists of a N-terminal cytoplasmic region, a membrane spanning domain and an exoplasmic C-terminal region accounting for ~50% of the protein length (Fig. 3A) (*38*, *39*). We have previously demonstrated the essentiality of the C-terminal domain of DivIC for both function and localization and the ability of the protein to interact with the cell wall in a WTA-dependent manner (*30*). DivIB is a PG-binding protein (*29*), and the requirement of the C-terminal domain for interaction with PBP1 and FtsW has been recently reported in *S. aureus* (*40*). To analyze the function of the C-terminal domain of FtsL, we expressed the complete or truncated versions of the protein lacking 10 (ΔV124-N133), 23 (ΔK111-N133), or 65 amino acids (ΔA69-N133) from the extracellular region using the pLOW-P*pcn* expression system. The presence of the truncated proteins was confirmed by Western blot (Fig. 3B). Expression of FtsL or FtsL ΔV124-N133 complemented Δ*ftsL*, allowing growth in agar in the absence of IPTG, and the expression of proteins did not affect the ability of the cells to grow in the presence of the inducer; however, only ~1% of Δ*ftsL* cells expressing ΔK111-N133 or ΔA69-N133 survived with no IPTG (Fig. 3C). Expression of FtsL lacking 40 amino acids (ΔA2-T41) from the N-terminal cytoplasmic domain (Fig. 3B) also complemented the loss of FtsL (Fig. 3C). Similarly, liquid cultures of FtsL-depleted cells expressing FtsL ΔK111-N133 or ΔA69-N133 lost viability after 1-hour growth, whereas the rest of the truncations allowed growth at the level of the FtsL-complemented cells (Fig. 3D). Although viability was not affected, the volume of Δ*ftsL* cells expressing FtsL ΔV124-N133 increased significantly compared to those complemented with FtsL. We found no volume change (Fig. 3, E and F) and cell stages of division (Fig. 3G) in FtsL ΔA2-T41–expressing cells.

### FtsL and DivIB control the activity of PBP3 and PBP4 to maintain morphology during cell division

**Fig. 4.**
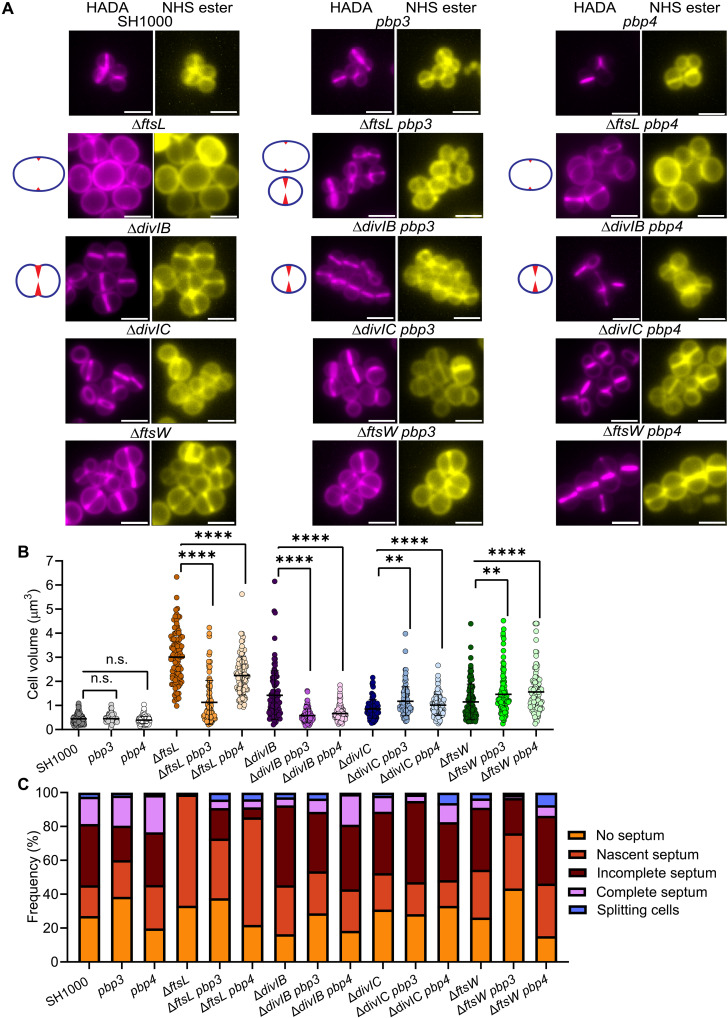
FtsL and DivIB control the activity of PBP3 and PBP4 during cell division. (**A**) Fluorescence microscopy of SH1000, *pbp3* (SJF5985), *pbp4* (SJF5989), Δ*ftsL* (SJF5665), Δ*ftsL pbp3* (SJF5984), Δ*ftsL pbp4* (SJF5988), Δ*divIB* (SJF3883) Δ*divIB pbp3* (SJF5982), Δ*divIB pbp4* (SJF5986), Δ*divIC* (SJF5450), Δ*divIC pbp3* (SJF6009), Δ*divIC pbp4* (SJF6010), Δ*ftsW* (SJF5761), Δ*ftsW pbp3* (SJF5983), and Δ*ftsW pbp4* (SJF5987). Cells were grown for 1 hour in the absence of IPTG (Δ*divIB* and its derivatives were grown for 2 hours). PG was labeled by 30-min incubation with HADA. Staining with NHS-ester 555 shows cell morphology. Images are average *z*-stack intensity projections. Scale bars, 2 μm. Images are representative of two independent experiments. Diagrams depict the most frequent phenotype; cell morphology (blue) and piecrust or incomplete septum (red) [based on data from (C)]. (**B**) Volume determined from cells stained with NHS-ester 555 [based on images in (A)]. Each circle indicates a single cell. *n* = 100 cells per sample. *P* values were determined by Mann-Whitney *U* tests (****P < 0.0001; **P < 0.0016). (**C**) Classification of cell stages of division based on HADA labeling [based on images in (A)]. *n* > 250 cells per sample.

The notable variations in size and morphology among cells lacking FtsL, DivIB (*29*), or DivIC (*30*) (Fig. 4, A and B) prompted us to speculate about these proteins playing distinct roles, not only in directing the migration of the members of the late division machinery to the division site but also in maintaining appropriate levels of PG synthesis and structural features of the peripheral cell wall. Because PBP3 and PBP4 play roles in peripheral PG synthesis, maintenance of cell morphology (*24*), and PG cross-linking (*13*, *25*, *41*), we introduced *Tn* insertions in the *pbp3* or *pbp4* genes (*27*) in the Δ*ftsL*, Δ*divIB*, and Δ*divIC* strains and analyzed images of cells grown without IPTG by fluorescence microscopy (Fig. 4A). The parental SH1000 and the *pbp3* and *pbp4* mutants have similar volumes (*x–* = 0.44, 0.45, and 0.4 μm^3^, respectively) (Fig. 4, A and B). However, we found an almost fourfold decrease in the volume of Δ*ftsL* in the absence of *pbp3* (Δ*ftsL pbp3*) (*x–* = 3.15 and *x–* = 0.81 μm^3^, respectively). A mutation in *pbp4* (Δ*ftsL pbp4*) produced a more modest but still significant decrease in volume (*x–* = 2.2 μm^3^) (Fig. 4, A and B). DivIB-depleted cells (*x–* = 1.5 μm^3^) had significantly decreased volume in the absence of either PBP3 or PBP4 (*x–* = 0.55 and 0.56 μm^3^, respectively). As previously reported (*30*), depletion of DivIC produces only a slight increase in the average volume with respect to SH1000 (*x–* = 0.75 and 0.48 μm^3^, respectively). Cells mutated in *pbp3* or *pbp4* showed a small but significant increase in the cellular volume (*x–* = 92 and 1.03 μm^3^, respectively) in the absence of DivIC (Fig. 4, A and B). As in cells lacking FtsL and DivIB, depletion of FtsW also impedes septal completion (Fig. 3G and fig. S4, A and B) (*24*) and produces an increase in cell volume (*x–* = 1.14 μm^3^) with respect to SH1000 (*x–* = 0.48 μm^3^) (Fig. 3F). The volume of FtsW-depleted cells increased in cells lacking *pbp3* or *pbp4* (*x–* = 1.46 and 1.56 μm^3^, respectively). Thus, in the absence of FtsL or DivIB, but not DivIC or FtsW, both PBP3 and PBP4 activities contribute to the increase in cell volume observed. We then analyzed FtsL- and DivIB-depleted cell populations in the presence and absence of PBP3 or PBP4 (Fig. 4C). Unlike FtsL-depleted cells where all cells showed either no septum or nascent septum, in cells lacking *ftsL* and *pbp3*, we observed that ~30% of the population was in stages ahead of the piecrust/nascent septum (i.e., incomplete septum, complete septum, and splitting cells). On the other hand, only ~12% of cells were in the late stages of septum formation in the absence of *ftsL* and *pbp4* (Fig. 4C). The absence of PBP3 in DivIB-depleted cells did not produce a significant increase in cells with complete septum or splitting, and the population of *divIC*- or *ftsW*-depleted cells lacking *pbp3* showed no increase in cells with complete septum or splitting. This suggests that FtsL and DivIB are regulators of PBP3 and PBP4, controlling their activity to maintain cell morphology during division by preventing runaway peripheral PG synthesis.

### Localization of MurJ to mid-cell partially depends on FtsL and is independent of DivIC and DivIB

**Fig. 5.**
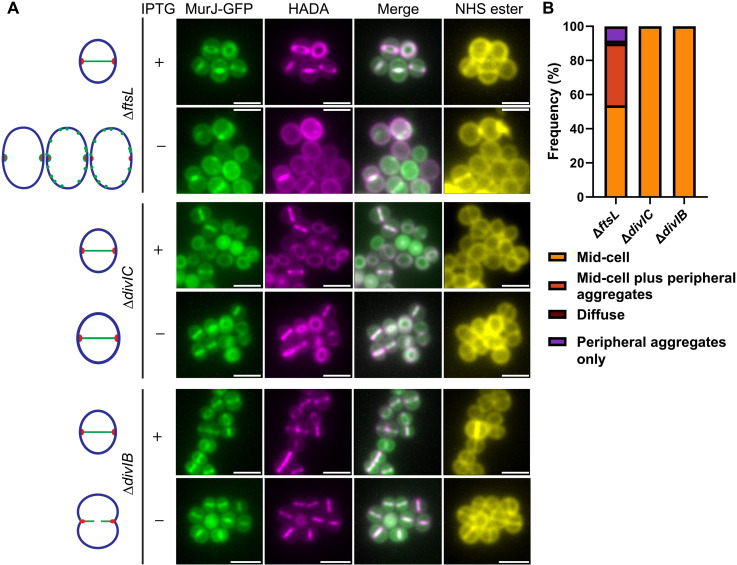
Localization of MurJ to mid-cell is partially affected by FtsL but is independent of DivIC and DivIB. (**A**) Fluorescence microscopy images of SH1000 Δ*ftsL* (SJF5957), Δ*divIC* (SJF5959), and Δ*divIB* (SJF5960) expressing MurJ-GFP were grown in the presence and absence of IPTG for 1, 2, and 2 hours, respectively. Incorporation of PG was followed for 5 min with HADA. Staining with NHS-ester 555 shows cell morphology. Images are average *z*-stack intensity projections. Scale bars, 2 μm. Images are representative of two independent experiments. Diagrams depict the patterns of the fluorescent fusion localization. Diagrams depict the most frequent patterns of the fluorescent fusion localization (mid-cell, mid-cell plus peripheral aggregates, or peripheral aggregates only) (green), the overall cell morphology (blue), and the mid-cell position (red). (**B**) Quantification of MurJ-GFP localization at mid-cell [based on images in (A)] for Δ*ftsL*, Δ*divIC*, and Δ*divIB* as above with localization defined as in Fig. 2D. Only cells showing incorporation of HADA at mid-cell were considered. *n* > 200 cells per sample.

MurJ is the only flippase in *S. aureus* able to translocate the precursor lipid II to the exoplasm for PG synthesis (*42*). In the MRSA strain COL, the requirement for the DivIB/DivIC/FtsL complex to recruit MurJ to the division site has been proposed, whereby the recruitment of MurJ forms a checkpoint for the initiation of PG synthesis during septum formation (*17*). As we have found distinct roles for FtsL/DivIB/DivIC, with differential levels of septum formation in their absence, we sought to determine their effects on MurJ recruitment to the division site. To accomplish this, we added HADA for 5 min to cells cultures depleted of FtsL, DivIC, or DivIB and expressing a MurJ fluorescent fusion (pLOW-*Ppcn-murJ*-*gfp*) to identify regions of PG synthesis (Fig. 5A). MurJ-GFP in the parental strain showed the expected, primarily septal localization as MurJ maps to the sites of PG synthesis (fig. S5, A and B) (*17*). In the Δ*ftsL* strain grown with IPTG, the same pattern of MurJ-GFP was maintained (Fig. 5, A and B, and fig. S5, A and B). We found that ~50% of Δ*ftsL* cells grown without an inducer also showed MurJ at mid-cell coinciding with HADA labeling; however, an additional ~40% also showed peripheral fluorescent aggregates, whereas ~10% showed peripheral aggregates with no fluorescence at mid-cell (Fig. 5, A and B). Although DivIB and DivIC are required for cell division, their depletion did not prevent the localization of septal MurJ-GFP at the site of PG synthesis (Fig. 5, A and B). Previous research has suggested that the arrival of MurJ at mid-cell is a checkpoint for nascent septum synthesis (*17*). We determined the effect of MurJ depletion on the localization of DivIB, DivIC, and FtsL using a *murJ* conditional lethal mutant constructed by replacing the native gene by a copy regulated by the P*spac* promoter (hereafter P*spac-murJ*). Cultures incubated in the presence of IPTG grew normally, but in the absence of the inducer, viability started to decline after 2 hours (fig. S9A). This coincided with the depletion of MurJ demonstrated by Western blot (fig. S9, B and C). The absence of the inducer led to a loss of plating efficiency of the P*spac-murJ* strain but not when complemented with a *murJ* expressing plasmid proving the essentiality of MurJ (fig. S9D). We used ADA-DA labeling (*32*) to demonstrate the role of MurJ in PG synthesis. At 2-hour incubation without the inducer, cells were still intact but only gave residual levels of ADA-DA incorporation (fig. S9E). Cessation of growth and division of P*spac-murJ* (fig. S9F) produced a modest increase in cell volume (fig. S9G) but did not coincide with cell cycle arrest at a particular stage (fig. S9, F and H). We then expressed GFP-fluorescent fusions of MurJ (as a control), DivIC, DivIB, and FtsL in the P*spac-murJ* strain to determine the role of MurJ in the localization of these proteins (fig. S10, A to D). After growing cultures for 2 hours with or without IPTG, we added HADA for 5 min to visualize PG synthesis and stained cells with *N*-hydroxysuccinimide (NHS)–ester 555 (to identify cell wall, due to poor HADA incorporation). DivIC, DivIB, and FtsL maintained septal localization despite the absence of MurJ (fig. S10B), suggesting MurJ-independent translocation of these proteins to the division site.

## DISCUSSION

*S. aureus* is a well-known pathogen and antibiotic resistant microorganism with spheroid morphology. Unlike rod-shaped counterparts that require a large number of PBPs and auxiliary proteins to perform, first elongation (regulated by the actin-like MreB proteins, the elongasome), and then cell division (initiated by the assembly of the Z-ring by the tubulin homolog FtsZ) (*43*, *44*), *S. aureus* lacks an elongasome and has only a modest set of proteins that efficiently pilot the complex process of cell division, turgor pressure maintenance, and size and shape regulation simultaneously (*2*). The positioning of the division site by the early division proteins sets in motion a highly coordinated process of PG synthesis that starts with the formation of the piecrust, a distinct ribbon-like structure that provides the foundation for septum formation (*18*). Here, we have demonstrated that FtsL is an essential player that controls the initiation of septation in *S. aureus*. In the absence of FtsL, cells form an aberrant nascent piecrust and are then unable to initiate the building of the septal plate. The stalled cells often also present more than one piecrust. They also experience uncontrolled peripheral growth producing enlarged, apparent lozenge-like cells that rapidly lose viability (Fig. 1 and figs. S1 and S2). Depletion of FtsL in *Escherichia coli* and *Bacillus subtilis* leads to long, aseptate filaments, and ultimately lysis (*36*, *45*). Although in *B. subtilis*, FtsL affected the migration of both DivIB and DivIC to the site of division (*36*), and in *E. coli*, depletion of FtsQ (DivIB) impaired septal localization of FtsL (*46*), we found that DivIC movement to mid-cell was severely affected in the absence of FtsL, whereas the localization of DivIB was independent of FtsL, and correspondingly FtsL was only marginally affected by DivIB in *S. aureus* (Fig. 2).

The formation of a trimeric complex of the orthologs FtsL, FtsB, and FtsQ in *E. coli* and *Streptococcus* (FtsL/DivIC/DivIB) has been previously demonstrated, but the function of the trimer is not well understood (*4*, *5*, *47*, *48*). There is clear evidence of a strong interdependence among the members of the DivIB/DivIC/FtsL trimer for stabilization, localization, and recruitment of PBP2B in *B. subtilis* and its counterpart, FtsI, in *E. coli*, the equivalent of PBP1 in *S. aureus*, to the division site (*36*, *46*, *49*–*53*). The C-terminal domain of DivIB is critical for interaction with PBP2B in *B. subtilis*, where a model of a quaternary complex of DivIB/PBP2B/FtsL/DivIC with DivIB, DivIC, and FtsL regulating the TP activity of PBP2B has been suggested (*6*, *54*). The stability of DivIC depends on FtsL, whereas DivIB protects FtsL from degradation (*6*, *36*), suggesting a regulatory mechanism. We have found that the loss of DivIB, DivIC, or FtsL has no effect on the stability of each other, or of other members of the divisome, in *S. aureus* [(*30*) and Fig. 2C]. The absence of DivIB or DivIC, leads to an increase in FtsL levels over time (*30*). These findings suggest that, although these proteins are highly conserved among bacteria (*4*–*6*), their dynamics have evolved to serve the specific needs of each microorganism to efficiently divide. A total of 73% of the *S. aureus* FtsL amino acids differ from those of the *Bacillus* protein. The C-terminal domain of *S. aureus* FtsL is not functional in *B. subtilis* (*55*).

FtsL is a bitopic membrane protein with an exoplasmic coiled-coil–like domain (*43*). The cytoplasmic and periplasmic domains of FtsL in *E. coli* are essential for function (*56*, *57*), and protein degradation requires the N-terminal cytoplasmic domain in *Bacillus* (*50*). We found that the N-terminal region of the *S. aureus* FtsL is functionally dispensable (Fig. 3). In agreement, the putative function of DivIC and DivIB in the regulation of PG synthesis at the septum seems to reside exclusively in their external domains because exogenous cytoplasmic and membrane bound segments did not modify the localization of DivIC to the division site (*58*). In *B. subtilis*, the extracytoplasmic domains of DivIC and DivIB are sufficient for both vegetative and sporulation division (*58*). In *S. aureus*, the C-terminal exoplasmic domain of DivIC, a cell wall–binding protein, is also essential for function and localization (*30*), whereas DivIB, a PG-binding protein, has been recently suggested to act as an activator of the PBP1-FtsW complex that depends on the C-terminal external region [the gamma domain; (*29*)] of the protein for activity and localization (*40*). Accordingly, we found that the functionality of FtsL resides in the C-terminal region of the exoplasmic domain FtsL (Fig. 3). Whether the FtsL function requires binding to cell wall components or is only dependent on protein-protein interactions remains elusive.

A synergy between EzrA, a regulatory protein that inhibits FtsZ assembly, and FtsL in regulating the FtsZ ring constriction has been suggested in *Bacillus* (*59*). The characterization of an FtsL mutant (E88K) in *E. coli* showed its ability to bypass the division proteins FtsK, FtsN, and ZipA and partly bypass FtsA. FtsN and FtsA play roles in constriction, suggesting involvement of FtsL in modulating divisome activation (*60*). Recent data from *E. coli* led to the suggestion that the cytoplasmic domain of FtsL recruits the FtsWI complex. The dimer is then activated by FtsN that requires the presence of FtsQBL and FtsA and contact with a periplasmic region of FtsL through FtsI (*61*). In *S. aureus*, the association of FtsA with FtsZ has been shown to promote FtsZ guanosine triphosphatase activity, suggesting a coordinated regulation of these proteins during cytokinesis (*62*). In Gram-positive bacteria ClpC, an adenosine triphosphate–dependent Hsp100/Clp chaperone plays a role in protein quality control and regulation of gene expression. FtsA has been identified as a ClpC substrate in *S. aureus*, pointing at a possible regulatory function (*63*). Because FtsN is absent in *S. aureus*, it is possible that FtsA and EzrA, previously shown to be able to interact with DivIC, DivIB, FtsL, PBP1, and PBP2 (*64*), can modulate the interface between the early and late division processes through interaction with the C-terminal domain of FtsL.

**Fig. 6.**
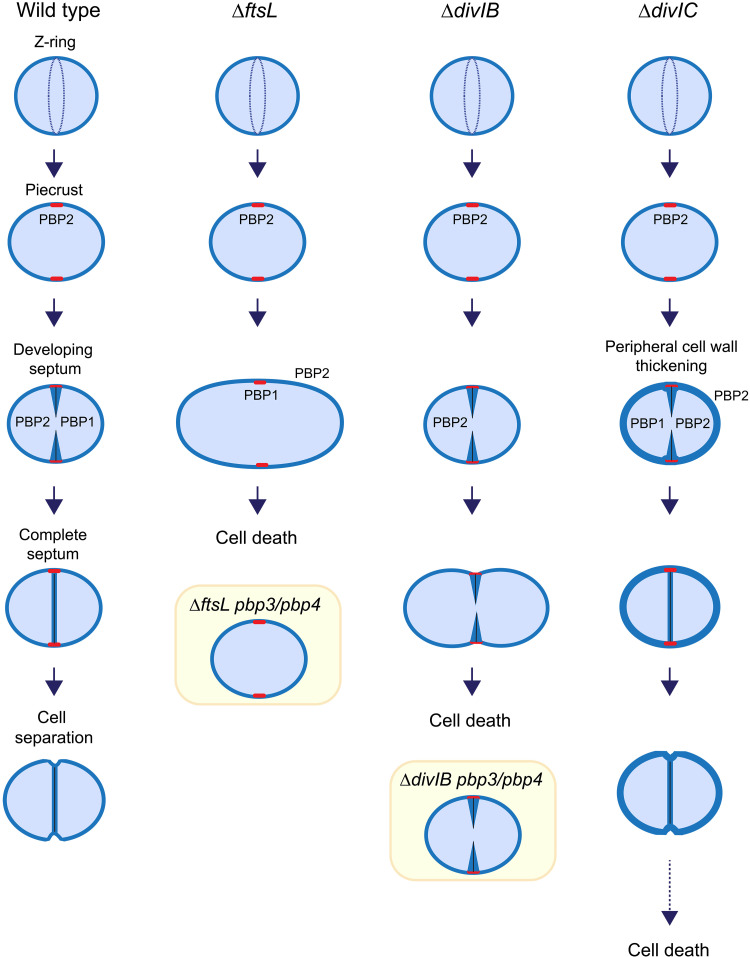
FtsL, DivIB, and DivIC have essential roles in septum development and cell morphology in *S. aureus* by controlling PBP localization and activity. In wild-type cells, septal synthesis starts with a PG foundation structure, the piecrust (red), which supports septal plate formation by PBP1 and then PBP2. In FtsL-depleted cells (denoted Δ*ftsL*), DivIC and PBP2 remain peripheral despite the presence of a piecrust. This causes a blockage on the formation of the septal plate. Also, the absence of FtsL results in deregulation of PBP3 and PBP4, causing elongation and volume increase (yellow square). These changes ultimately lead to cell death. In DivIB-depleted cells (Δ*divIB*) (*29*), DivIC and PBP2 are localized to mid-cell, allowing the progression of PG synthesis inward; however, the septal plate cannot be completed. PBP1 does not localize to the division site in the absence of DivIB, suggesting the involvement of PBP1 in septum completion. In DivIB-depleted cells, PBP3 and PBP4 (yellow square) are deregulated, causing morphological defects and volume increase. These changes cause cell death and lysis. In DivIC-depleted cells, PBP2 mainly remains peripheral, causing abnormal thickening of the surrounding cell wall (*30*). DivIC-depleted cells increase in volume in a PBP3/PBP4-independent way. These changes eventually lead to cell death.

The PG synthase PBP2 plays a major role in *S. aureus* (*65*), its recruitment for the bulk septal synthesis is facilitated by the presence of its substrate (*66*), and it has been proposed to interact with several division proteins including DivIC, DivIB, and FtsL (*64*). As previously mentioned, DivIC is required for septal localization of PBP2, which makes DivIC an essential regulator of septum formation (*30*). Herein, we show that FtsL is required for the migration of DivIC and PBP2 to the division site (Fig. 2); however, because DivIC-depleted cells are still able to produce septa, but loss of FtsL blocks the division process at an early stage (after piecrust formation), we conclude that FtsL is an early checkpoint in septal development, enabling the recruitment of DivIC that, in turn, facilitates the movement of PBP2 to the division site for septal plate formation (Fig. 6). The consequent development of the septal plate requires PBP2 plus the PBP1-FtsW complex (*18*); however, neither PBP1 nor FtsW require FtsL translocation to the division site (Fig. 2) and PBP1 is only partially dependent on DivIC (*30*). We have proposed that PBP1 is responsible for the PG rings that are made as the initial phase of septal plate formation (*11*, *19*, *27*) and subsequently the bulk of the septal PG mesh is filled out by PBP2. Thus, FtsL may be required for the activation of PBP1 activity or that the piecrust produced by FtsL-depleted cells does not form a useful foundation for septal PG ring formation. As the piecrust is made in FtsL-depleted cells and is not dependent on PBP1 activity (*11*, *27*), we suggest that it is made by PBP2 directed by FtsZ-dependent early cell division machinery. The double piecrusts sometimes observed in FtsL-depleted cells (Fig. 1E) may be due to the early division machinery not being disassembled with continued synthesis of abortive septa.

DivIB is essential in *S. aureus*, and its depletion produces a significant increase in cell size and results in aberrant phenotypes, including the initiation of several parallel septa, swelling of the cell (“hamburger” cells), and lysis (*29*). In the absence of DivIB, both FtsZ and EzrA are recruited to the division site and the first steps of septal formation are not affected; however, completion of the septal plate is blocked (fig. S2) (*29*). We observe a >45% decrease in PBP1 recruitment to the septum in DivIB-depleted cells (fig. S6). DivIC partially requires DivIB for recruitment to mid-cell but not vice versa (*30*), whereas PBP2 is DivIB independent. Thus, we suggest that DivIB plays a role as a septum formation checkpoint by facilitating the localization, and perhaps the activity, of PBP1. This causes profound consequences for the architecture of the septal plate, impeding its closure and leading to cell death (Fig. 6). It is possible that DivIB, through interaction with PG (*29*), senses changes in the architecture of the developing structure and, by interaction with PBP1, facilitates septal synthesis by the PBP1-FtsW complex (Fig. 6).

One of the characteristics associated with cells lacking DivIC, DivIB, or FtsL is the increase in cell volume, but overall morphologies are specific to each of them. DivIC-depleted cells show a moderate increase in volume (Fig. 4) (*30*), abnormal holes in the inner side of the septa, and thickening of the peripheral cell wall. This is attributed to the inability of PBP2 to migrate toward the septum in the absence of DivIC while remaining active at the periphery (*30*). Contrary, the much more severe morphological changes due to the loss of FtsL or DivIB seem to be linked to deregulation of the nonessential PBP3 and PBP4 activities (Fig. 4). An alternative explanation could be that stalling septum formation provides a pool of PG precursors that could be used for peripheral synthesis, resulting in the observed volume increase. Depletion of FtsW leads to a clear septum formation defect, and this does not result in a PBP3- or PBP4-dependent increase in cell volume (Fig. 4). This reveals a regulatory mechanism by FtsL and DivIB that ensures not only hierarchical translocation of the division machinery to the division site but also a tight control of the peripheral PG synthetic activity during the cell cycle (Fig. 6).

A compelling amount of evidence supports independent and hierarchical functions for FtsL, DivIB (*29*, *40*), and DivIC (*30*) in *S. aureus.* These proteins sequentially coordinate the late stages of cell division by modulating both the localization of PBP2 and PBP1 to the division site and their subsequent activities. This positive regulation is also correlated with a negative regulation of PBP3 and PBP4 at the cell periphery, ultimately guaranteeing an efficient process of cell division and maintenance of cell morphology.

A model for *S. aureus* has been proposed in which MurJ recruitment to the division site by the DivIB/DivIC/FtsL trimer redirects the PG synthesis to the septum by ensuring that translocation of lipid II only occurs at the division site, facilitating the movement of the PBPs to mid-cell (*17*). We find that MurJ is required for PG synthesis at all stages of the cell cycle and does not form a distinct cell division checkpoint. PG synthesis during septum formation occurs as a series of architectural and biochemical events, which mark clear and important transitions to allow division to proceed. Initially, the piecrust is formed, which we hypothesize is the result of PBP2 activity, driven by the FtsZ ring complex. The piecrust forms the foundation for the septal plate that is initially formed as a thin layer of PG, with a characteristic ring architecture of long glycan strands. The rings are proposed to be made by PBP1/FtsW, and this is supported by the FtsW trajectory (*11*, *40*). The ring structure leads to closing of the septal annulus but with an initial bowed structure. Around the rings, the bulk of the septum is formed by PBP2, which synthesizes a fine mesh as found around the inside of the cell periphery. FtsL/DivIB/DivIC recognizes these changing structures and direct PG synthesis across these transitions to allow cell division to occur. Given the high degree of conservation of these three regulatory proteins, insight into *S. aureus* cell division provides a framework for further understanding across bacteria.

## MATERIALS AND METHODS

### Bacterial strains and growth conditions

Lists of strains, plasmids, and oligonucleotides are provided in tables S1, S2, and S3, respectively. Strains were cultured at 37°C with aeration. *E. coli* was grown in Luria-Bertani (LB) broth or agar plus ampicillin (100 μg/ml). *S. aureus* strains were cultured in tryptic soy broth (TSB) or agar (TSA) (Bacto, BD 21182). Erythromycin (5 μg/ml) plus lincomycin (25 μg/ml) (Ery/Lin), chloramphenicol (Cm) (10 μg/ml), tetracycline (Tet) (5 μg/ml), and kanamycin (Kan) (50 μg/ml) or trimethoprim (Tm) (10 μg/ml) were added when required.

### Construction of plasmids and strains

*E. coli* NEB5α strain was used for construction of the indicated vectors (table S2) according to previously described methods (*67*, *68*). Restriction-deficient *S. aureus* strain RN4220 was used for introduction of plasmids by electroporation followed by phage transduction into *S. aureus* SH1000 and its derivatives as formerly described (*69*). All chromosomal modifications and plasmid constructs were confirmed by sequencing (Source BioScience).

### SJF5665 (*ftsL* conditional lethal strain, Δ*ftsL*)

A copy of the *ftsL* gene under control of the P*spac* promoter (pKASBAR-*ftsL*) was introduced into the *geh* chromosomal locus. For the assembly of pMAD-U-*tet*-D*ftsL*, SH1000 genomic DNA was used as a template to amplify 964 bp upstream (U*ftsL*) (primers 109 and 110) and 1000 bp downstream including the 3′ sequence of *ftsL* from nucleotide 223 (D*ftsL*) (oligos 113 and 114) to avoid disrupting the promoter of the subsequent gene (*pbp1*) (fig. S1A). The tetracycline resistance cassette from pOB (*20*) was amplified using oligos 111 and 112. The polymerase chain reaction (PCR) products were cloned into pMAD digested with Bgl II and Bam HI according to the NEBuilder HiFi DNA Assembly Cloning Kit (E5520S) instructions. pMAD-U*-tet*-D*ftsL* was inserted in the chromosome by single crossover recombination followed by excision of the pMAD derivative by double crossover as described previously (*70*). To provide a tight control of the P*spac*-*ftsL* system, a plasmid constitutively expressing *lacI* (pGL485) was introduced by transduction.

### SJF5761 (*ftsW* conditional lethal strain, Δ*ftsW*)

The assembly of the *ftsW* gene under control of the P*spac* promoter was achieved by amplifying P*spac* (oligos 215 and 216) from pKASBAR-*ftsL* and *ftsW* from SH1000 genomic DNA (oligos 217 and 218). The PCR products were inserted into previously digested pKASBAR according to the NEBuilder HiFi DNA Assembly Cloning Kit (E5520S) instructions. For the assembly of pMAD-U-D*ftsW*, SH1000 genomic DNA was used as a template to amplify 782 bp upstream (U*ftsW*) (oligos 219 and 220) and 777 bp downstream (D*ftsW*) (oligos 221 and 222) (fig. S3A). The PCR products were cloned into pMAD previously digested according to the NEBuilder HiFi DNA Assembly Cloning Kit (E5520S) instructions. pMAD-U-D*ftsW* was inserted in the chromosome by single crossover recombination followed by excision of the pMAD derivative by double crossover as described previously (*70*). A plasmid constitutively expressing *lacI* to provide a tight control of the P*spac*-*ftsL* system (pGL485) was introduced by transduction.

### SJF6065 (*murJ* conditional lethal strain, Δ*murJ*)

Primers 785 and 786 were used to amplify the 5′ region of *murJ* using COL genomic DNA. The PCR product and the pMUTIN plasmid (*71*) that carries a P*spac* promoter were digested with Hind III and Kpn I and ligated to render pMUTIN-HA-*murJ*. The construct was inserted into the SH1000 chromosome by single crossover recombination. A plasmid constitutively expressing *lacI* to provide a tight control of the P*spac*-*murJ* system (pGL485) was introduced by transduction. For *murJ* complementation, *murJ* was amplified from COL genomic DNA using oligos 916 and 917. The PCR product was digested with Nhe I and Bam HI and then ligated into the pKK30 plasmid to generate pKK30-*murJ*, and the construct was introduced to the Δ*murJ* strain by phage transduction.

### Plating efficiency

Strains were incubated in TSB containing Cm (plus Ery/Lin when required) and 1 mM IPTG to exponential phase [optical density at 600 nm (OD_600nm_) of 0.4]. Cells were washed twice with TSB by centrifugation. The pellets were resuspended in 0.5 ml of TSB followed by dilution in TSB to an OD_600nm_ of 0.5. Decimal serial dilutions of samples were inoculated in TSA containing antibiotic(s) with and without 1 mM IPTG. Plates were incubated for 24 hours. Plating efficiency was calculated as colony-forming units per volume (CFU/ml) in the presence and absence of IPTG (*27*).

### Cell growth and survival curves

Strains were incubated in TSB containing Cm and 1 mM IPTG to reach exponential phase (OD_600nm_ of 0.4). Cells were washed twice with TSB by centrifugation and used to inoculate fresh medium to an OD_600nm_ of 0.05 in the presence of Cm with or without 1 mM IPTG. For growth, changes in OD_600nm_ were monitored every hour for 5 hours. For survival, decimal serial dilutions of samples taken every hour for 5 hours were inoculated in TSA containing an antibiotic and 1 mM IPTG. Plates were incubated for 24 hours. Plating efficiency was calculated as colony-forming units per volume (CFU/ml) in the presence and absence of IPTG (*27*, *30*).

### Expression of fluorescent fusions

To assess localization of fluorescent fusions, cultures of SH1000 and the conditional lethal strains were grown in TSB containing Cm, Ery (or Kan when indicated), and 1 mM IPTG to exponential phase (OD_600nm_ of 0.4). Cells were washed once by centrifugation-resuspension with TSB and inoculated in fresh medium plus antibiotics with or without IPTG at an OD_600nm_ of 0.05 (*27*, *29*, *30*). A Cd-inducible promoter was used to limit the expression of GFP-PBP1 and GFP-FtsL (P*cad-gfp-pbp1* and P*cad-gfp-ftsL*, 1 and 0.2 μM CdCl_2_, respectively) (*72*). GFP-PBP1 expressed to higher levels caused cell toxicity, whereas GFP-FtsL accumulated in the cytoplasm, hampering localization analysis. Cells were incubated for further 1 or 2 hours prior to harvesting for fixation.

### Cell growth and labeling with d-amino acids and NHS-ester for fluorescence microscopy

Strains were incubated in TSB containing Cm (Ery/Lin or Kan were also added for strains carrying fluorescent fusion vectors) and 1 mM IPTG to reach exponential phase (OD_600nm_ of 0.4). Cells were washed twice with TSB by centrifugation and used to inoculate fresh medium to an OD_600nm_ of 0.05 in the presence of Cm with or without 1 mM IPTG. For labeling of nascent PG, 1 mM HADA (Bio-Techne, 6647) or 500 μM ADA-DA was added to cultures for the indicated times (i.e., 5, 10, or 30 min) prior to the culture incubation time completion (i.e., 1 or 2 hours) (*27*). Cells were washed with phosphate-buffered saline (PBS) by centrifugation. When indicated, cells were stained with NHS-ester 555 (Invitrogen, A20009) in PBS at 8 μg/ml for 5 min and fixed. Cells treated with ADA-DA were further labeled with Fluor-488 Alkyne (Merck, 761621) by the click reaction [copper(I)-catalyzed alkyne-azide cycloaddition] (for fluorescent tagging of the ADA-DA azide group) using the Click-iT Cell Reaction Buffer Kit (Invitrogen, C10269) following the manufacturer’s instructions (*27*, *30*, *37*).

### Cell labeling with Nile red

Nile red (Sigma-Aldrich, 72485) (5 μg/ml) was added to cells resuspended in PBS for 5 min at room temperature. Cells were centrifuged and washed with water prior to fixing (*24*).

### Cell labeling with Bocillin

Bocillin (fluorescent penicillin) (Thermo Fisher Scientific, B13233) (0.5 μg/ml) was added to cell cultures followed by 5-min incubation at 37°C with aeration. Cells were washed twice with PBS prior to fixing.

### Cell fixation and slide mounting for optical microscopy

Cells were resuspended in 2% (w/v) paraformaldehyde (PFA) and incubated for 30 min at room temperature. PFA was removed by washing with water, and cells were mounted onto poly-l-lysine–coated slides using Slow Fade Diamond mounting medium (Thermo Fisher Scientific, S36967) prior to imaging (*27*, *30*, *37*).

### Wide-field epifluorescence microscopy

Images were acquired using a Nikon Ti inverted microscope equipped with a Lumencor Spectra X light engine and a 100x PlanApo (1.4 numerical aperture) oil objective (1.518 refractive index oil). An Andora Zyla sCMOS camera was used for detection.

### OMX microscopy (structured illumination microscopy)

Coverslips (high precision, 1.5H, 22 ± 22 mm, 170 ± 5 mm; Marienfeld, 0107052) were immersed in 1 M KOH, sonicated for 10 min, and rinsed with water prior to incubation in poly-l-lysine solution for 30 min. Coverslips were washed and dried with pressurized air before use. Fixed cells were dried onto coverslips with pressurized air prior to mounting on slides using Slow Fade Diamond mounting medium (Thermo Fisher Scientific, S36967). Structured illumination microscopy (SIM) was performed in a v4 DeltaVision OMX 3DSIM system fitted with a Blaze module (Applied Precision, GE Healthcare, Issaquah, USA) with laser illumination. Images were acquired in five phase shifts and three angles for each slice with 0.125-nm *Z*-steps. Softworx software (GE, Healthcare) was used for reconstruction with optical transfer function optimization for immersion oil 1.516.

### Cell volume determination

Cell volume was determined as previously described (*73*). Measurements of the long and short axis of cells were performed using the software Fiji (*74*), and the volume was calculated according to a prolate spheroid shape using the formula

$$V=\frac{4}{3}\pi ab^{2}$$

*V* is the volume, and *a* and *b* represent the radii for the long and short axis, respectively.

### Quantification of the localization of fluorescent fusions

Images from *z*-stack intensity projections were analyzed. To determine the localization of GFP-tagged proteins, only cells showing incorporation of PG at mid-cell (HADA- or ADA-DA–derived fluorescence) were analyzed. Cells were then classified according to the position of green fluorescence (mid-cell or peripheral fluorescence). When loci of higher intensity were observed, cells were classified as containing protein aggregates. GFP-fusions showing fluorescence but no specific localization were classified as “diffuse.”

### Transmission electron microscopy

Cell treatment for electron microscopy was performed as previously described (*75*). Cell pellets were fixed in 2.5% (w/v) glutaraldehyde for 12 hours at 4°C followed by washing with PBS. For secondary fixation, samples were resuspended in 2% (w/v) aqueous osmium tetroxide (Merck, 75632) and incubated for 2 hours at room temperature. After washing with PBS, samples were dehydrated by 15-min incubation in increasing concentrations of ethanol [75 (v/v), 95 (v/v), and 100%] followed by treatment with propylene oxide (Merck, 540021) to complete dehydration. To allow infiltration, a mix of propylene oxide and Epon resin (TAAB Laboratories, E208) (1:1) was used to incubate the samples overnight at room temperature. After resin was removed, and propylene oxide evaporated at room temperature, samples were incubated two times with Epon resin for 4 hours each period. Fresh resin was used to embed cells, and polymerization was achieved at 60°C for 48 hours. An Ultracut E Ultramicrotome (Reichert-Jung) was used to obtain thin sections (80 nm, approximately) that were mounted onto 200-square mesh copper TEM grids (Merck, G4776-1VL) pretreated with pyroxylin (1.5%) (w/v, in amyl acetate) film. Sections were incubated in aqueous uranyl acetate (3%) (w/v) for 30 min, washed with water, and stained for 5 min with Reynold’s lead citrate (*76*). Images were obtained using an FEI Tecnai T12 Spirit transmission electron microscope operating at 80 kV. A Gatan Orius SC1000B bottom-mounted charge-coupled device camera was used to record the images.

### Extraction and purification of cell wall (sacculi)

Extraction was performed as previously described (*18*, *75*). Cells grown in the presence or absence of IPTG were centrifuged (5 min, 17,000*g*), and the resulting pellets were boiled for 15 min to kill the cells. Samples were then resuspended in PBS and added to lysing matrix tubes containing 0.1-mm silica beads (Lysing Matrix B, MP Biomedicals, 116911050-CF). Cells were broken in a FastPrep-24 homogenizer (10 cycles of 30 s each, speed of 6.5 m/s). Broken cells were separated from the lysing matrix by low-speed centrifugation (5 min, 2400*g*), and sacculi were harvested by high-speed centrifugation (15 min, 220,000*g*). Sacculi were then resuspended 4% (w/v) SDS and boiled for 30 min followed by centrifugation and resuspension in 50 mM tris-HCl (pH 7.5) containing 3% (w/v) SDS, 1.25 mM EDTA, and 50 mM dithiothreitol. Samples were boiled for a further 30 min and washed repeatedly with water to remove SDS. Samples were then resuspended in tris-HCl (50 mM pH 7.5) containing pronase (2 mg/ml) and incubated for 90 min at 60°C. Pure cell walls resuspended in high-performance liquid chromatography (HPLC)–grade water, washed three times by centrifugation and resuspension in water before storage at 4°C until use.

### AFM imaging

Aliquots of extracted purified cell walls were immobilized on a Cell-Tak–coated mica substrate (Corning Netherlands) using the immobilization protocol as previously described (*19*). Briefly, prior to the PG immobilization, 180 μl of Cell-Tak solution [6 μl of Cell-Tak (1.05 mg/ml) + 3 μl of 1 M NaOH +171 μl of 100 mM NaHCO_3_ (pH 8)] was incubated on a freshly cleaved mica for 30 min. The substrate was rinsed three times with HPLC water and blow-dried with nitrogen flow. Afterward, 40 μl of diluted purified cell wall suspension was incubated on the Cell-Tak–coated surface for 90 min. The immobilized sample was rinsed three times with HPLC water and blow-dried. Imaging buffer [10 mM Tris, 10 mM MgCl_2_, and 200 mM KCl (pH 8)] (300 μl) was added onto the sample for AFM high-resolution imaging. AFM high-resolution images were captured under buffering conditions using peak force tapping mode in Dimension FastScan AFM with FastScanD cantilevers (Bruker, Santa Barbara, USA). The scanning and feedback parameters used are peak force setpoint of 1 to 2 nN, peak force amplitude of 80 to 100 nm, in-house spring constant of 0.1 to 0.14 N/m, scan rate of 0.5 to 1 Hz, and pixels of 512 to1024. All images were processed using either Gwyddion 2.55 (Department of Nanometrology, Czech Metrology Institute, Czech Republic) or Nanoscope analysis software (Bruker, Santa Barbara, USA).

### FtsL topological model

The hypothetical topological model of FtsL was obtained using Gapped BLAST and PSI-BLAST (*77*).

### Whole-cell lysate preparation for Western blot

Cell cultures were grown according to the procedure described above for “cell growth and survival.” Cultures were taken at the indicated times after incubation in the presence and absence of IPTG and chilled in ice. Cells were washed twice by centrifugation-resuspension with PBS and resuspended in 0.5 ml of ice-cold TBSI [50 mM tris-HCl (pH 7.0)] containing protease inhibitors (Merck, 11836170001). Cell suspensions contained in lysing matrix tubes (0.1-mm glass beads, MP Biomedicals, 6911100) were broken using an MP Biomedicals FastPrep-24 homogenizer. Glass beads and unbroken cells were separated from the supernatant (whole-cell lysate) by low-speed centrifugation (5 min, 2500*g*) at 4°C (*27*, *30*).

### Protein quantification

Protein content was determined by using the Pierce BCA Protein Assay Kit (Thermo Fisher Scientific, 23227) according to the manufacturer’s instructions.

### Western blot

Proteins from whole-cell lysates (10 μg per sample) were separated by SDS–polyacrylamide gel electrophoresis (12%, w/v) and transferred to activated polyvinylidene difluoride membranes. Skimmed milk (5%, w/v) dissolved in TBST [20 mM tris-HCl (pH 7.6), 17 mM NaCl, and 0.1% (v/v) Tween 20] was used to block membranes (1 hour, at room temperature) and for incubation with primary antibodies (1:1000 dilution) overnight at 4°C followed by incubation with goat anti-rabbit immunoglobulin G (IgG) conjugated to horseradish peroxidase (1:10,000 dilution; Sigma-Aldrich, 12-348) (1 hour, at room temperature). Clarity Western ECL Substrate (Bio-Rad, 1705061) was used for detection according to the instructions. Chemodetection was performed in a Syngene G:BOX Chemi XX9 detector. Signal quantification from Western blots was obtained by measuring the signal intensity of three independent repeats using ImageJ Fiji software (*30*).

### Antibodies

Polyclonal antibodies were generated from rabbits immunized with small synthetic peptides: MAVEKVYQPYDEQVYC and CNDNVKVVRSNGEAKN for anti-FtsL antibodies (*30*), RKKQQQK-VDIRRQFN and VIGKDINGSKSWINL for anti-FtsW, and RKRK-HNIDRMVESDY and GHDPNHDGSRLLFYY for anti-MurJ. Affini-ty antigen-specific IgG purification was performed (Eurogentec). Anti-FtsZ (*29*) and anti-DivIC (*30*) polyclonal antibodies were obtained from rabbits immunized with purified His-tagged recombinant *S. aureus* FtsZ and DivIC (BioServ).

### Statistical analysis

Statistical analysis was performed in GraphPad Prism version 9.0. Sample size, the number of technical and biological repeats, and statistical tests are mentioned in the figure legends. Mann-Whitney *U* tests were applied to determine statistical differences in cell volumes and fluorescence rates derived from fluorescence microscopy images. Two-tailed unpaired *t* test was applied to compare cell viability data.

## Supplementary Materials

### This PDF file includes:

Figs. S1 to S10 Tables S1 to S3 References
