# Supplementary material for: Control of morphogenesis during the Staphylococcus aureus cell cycle
Source: Sci Adv. 2025 Apr 11;11(15):eadr5011. doi: 10.1126/sciadv.adr5011 (PMC11988411; doi:10.1126/sciadv.adr5011)
Supplement: Supplementary file 1 — Figs. S1 to S10 Tables S1 to S3 References [file sciadv.adr5011_sm.pdf]

Science Advances
AAAS

Supplementary Materials for

Control of morphogenesis during the *Staphylococcus aureus* cell cycle

Mariana Tinajero-Trejo *et al.*

Corresponding author: Simon J. Foster, s.foster@sheffield.ac.uk

Sci. Adv. **11**, eadr5011 (2025)
DOI: 10.1126/sciadv.adr5011

This PDF file includes:

Figs. S1 to S10
Tables S1 to S3
References

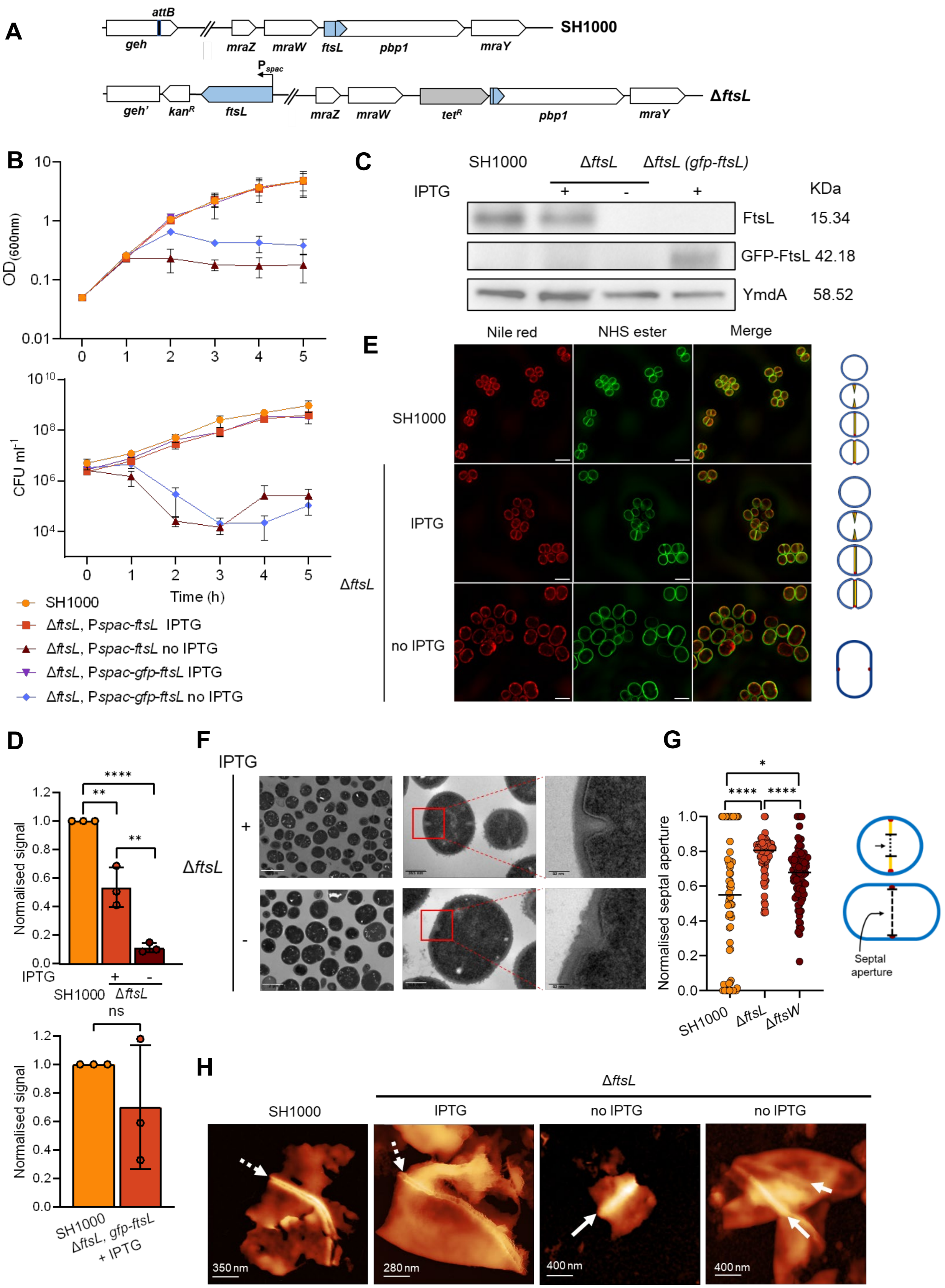
A
attB
geh
mraZ
mraW
ftsL
pbp1
mraY
SH1000
Pspac
geh'
kanR
ftsL
mraZ
mraW
tetR
pbp1
mraY
ΔftsL
B
OD(600nm)
CFU ml-1
Time (h)
SH1000
ΔftsL, Pspac-ftsL IPTG
ΔftsL, Pspac-ftsL no IPTG
ΔftsL, Pspac-gfp-ftsL IPTG
ΔftsL, Pspac-gfp-ftsL no IPTG
C
SH1000
ΔftsL
ΔftsL (gfp-ftsL)
IPTG
KDa
FtsL 15.34
GFP-FtsL 42.18
YmdA 58.52
E
Nile red
NHS ester
Merge
SH1000
IPTG
no IPTG
ΔftsL
D
Normalised signal
IPTG
SH1000
ΔftsL
ns
SH1000
ΔftsL, gfp-ftsL + IPTG
F
IPTG
ΔftsL
G
Normalised septal aperture
SH1000
ΔftsL
ΔftsW
Septal aperture
H
SH1000
ΔftsL
IPTG
no IPTG
no IPTG
350 nm
280 nm
400 nm
400 nm

Supplementary figure 1. FtsL is essential for growth, viability in *S. aureus.*

A. Genetic arrangement of the isogenic (SH1000) and the P*spac-ftsL* conditional lethal strain (Δ*ftsL*, SJF5665). **B.** Growth (OD_{600nm}) (top) and viability (CFU ml^{-1}) (bottom) of SH1000, P*spac-ftsL* and P*spac-gfp-ftsL* (SJF5666) conditional lethal strains in the presence and absence of IPTG. Means and standard deviations of three independent experiments are shown. **C.** Western blot of whole cell lysates of SH1000 P*spac-ftsL* and P*spac-gfp-ftsL* grown for 1 h in the presence or absence of IPTG. FtsL was detected using anti-FtsL antibodies. YmdA detection with anti-YmdA was used as loading control. **D.** Western blot signal quantification (based on C). Results are the average and standard deviation of three independent repeats. *P* values were determined by two-tailed unpaired *t*-test (left to right, ****, $P < 0.0001$; **, $P = 0.007$ and 0.0045). **E.** Structured Illumination Microscopy (SIM) images of SH1000 and Δ*ftsL* cells grown for 1 h with or without IPTG. Cell membranes were stained with Nile red and NHS-ester 555 was used to label the cell envelope. Images are average z stack intensity projections. Scale bars, 2 µm. Images are representative of two independent experiments. **F.** TEM of Δ*ftsL* (SJF5665) grown for 1 h with or without IPTG. Scale bars 2 µm (left) and 365 nm (right). **G.** Determination of septal aperture [based on images in F (SH1000 and Δ*ftsL*) and on Supplementary fig. 4B (Δ*ftsW*, SJF5761)]. Diagrams depicts how apertures were measured. Each circle indicates septal aperture in a single cell. $n \geq 70$ cells per sample. *P* values were determined by Mann–Whitney U tests (****, $P < 0.0001$; *, $P = 0.0443$). **H.** Atomic Force Microscopy (AFM) descriptive comparison between SH1000 and Δ*ftsL* (SJF5665) grown for 1 h with or without IPTG. Images show the structural features of piecrust in SH1000 and cells expressing FtsL (dotted arrows), and in FtsL-depleted cells with single and double septa (solid arrows).

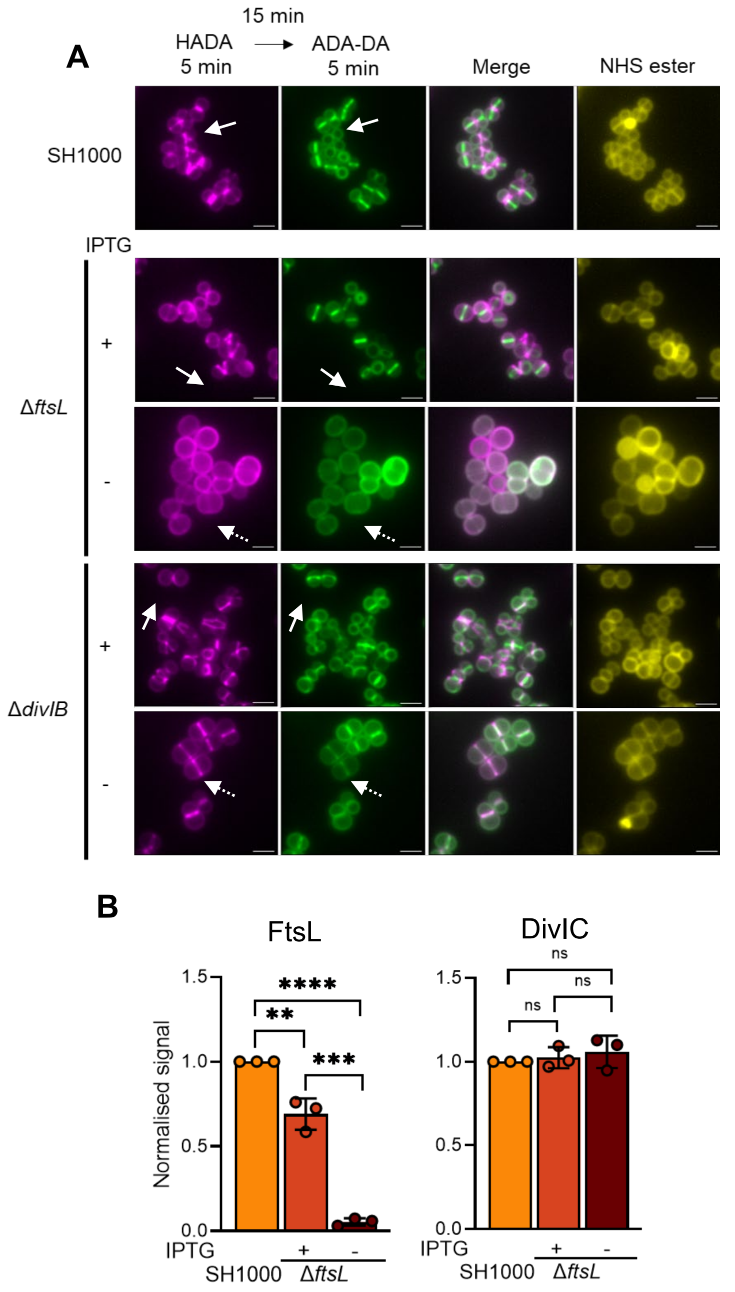

Supplementary figure 2. FtsL is essential for the initiation of septal plate whereas DivIB is required for closure.

A. Fluorescent microscopy images of Δ*ftsL* (SJF5665) and Δ*divIB* (SJF3883) cells grown with or without IPTG for 1 and 2 h respectively. SH1000 is shown as a control. Cells were further incubated with HADA for 5 min followed by 15 min incubation in fresh medium and ADA-DA was added for 5 min. Solid arrows indicate cells progressing to the next stages of cell division. Dotted arrows show cells with no further progression. NHS-ester 555 was

used to label the cell envelope. Images are average z stack intensity projections. Scale bar, 2 µm. Images are representative of two independent experiments. **B.** The stability of DivIC is not affected in the absence of FtsL. Signal quantification from Western blots of whole cell lysates of SH1000 and Δ*ftsL* (SJF5665) grown for 1 h in the presence or absence of IPTG (see Fig. 2C). Anti-FtsL and anti-DivIC antibodies were used for detection. Results are the average and standard deviation of three independent repeats. *P* values were determined by two-tailed unpaired *t*-test (****, $P < 0.0001$; ***, $P = 0.0003$; **, $P = 0.0044$).

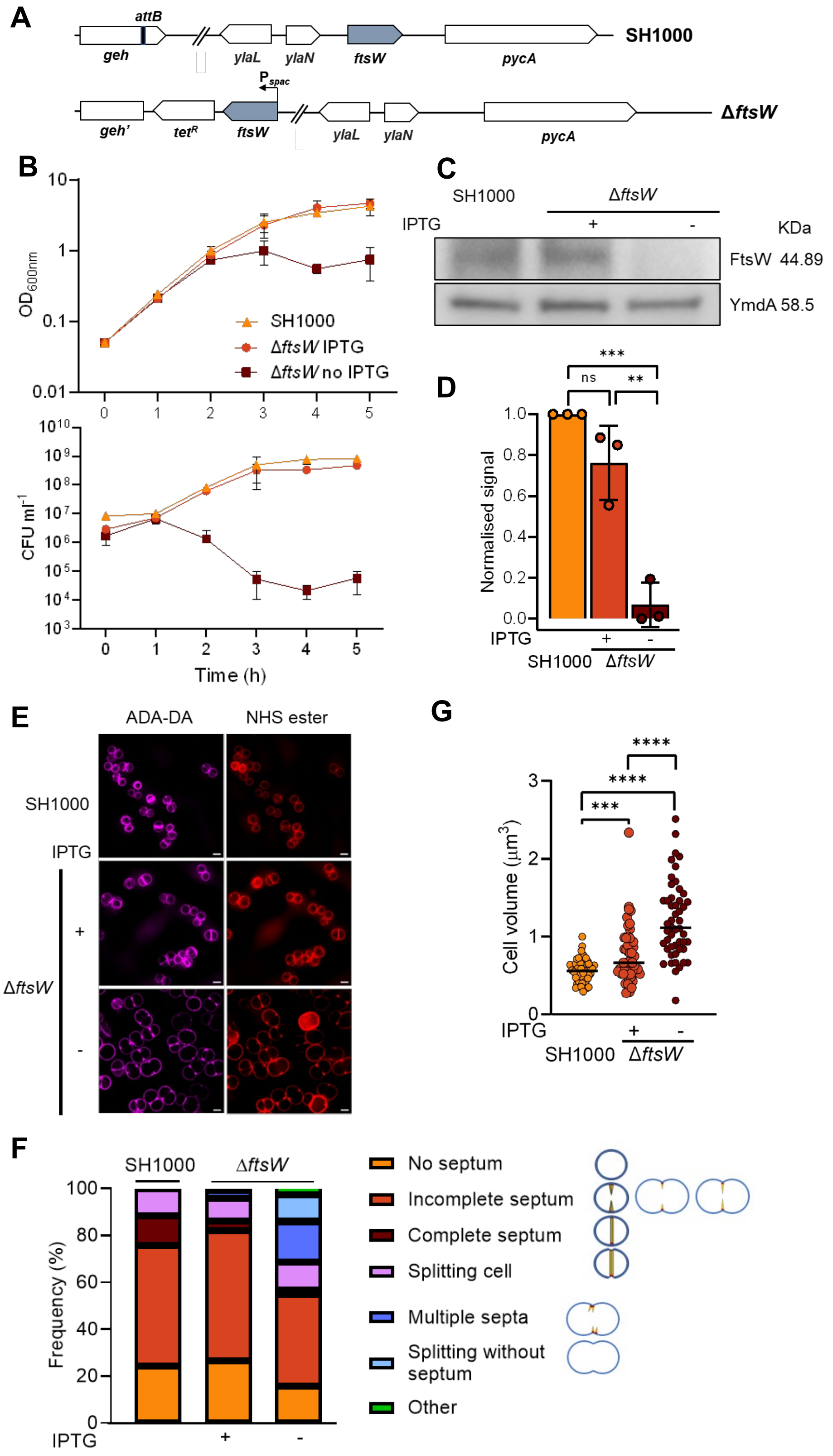
A
attB
geh
ylaL
ylaN
ftsW
pycA
SH1000
P spac
geh'
tetR
ftsW
ylaL
ylaN
pycA
ΔftsW
B
OD600nm
SH1000
ΔftsW IPTG
ΔftsW no IPTG
CFU ml-1
Time (h)
C
SH1000
ΔftsW
IPTG
+
-
KDa
FtsW 44.89
YmdA 58.5
D
Normalised signal
***
ns
**
IPTG
SH1000
ΔftsW
E
ADA-DA
NHS ester
SH1000
IPTG
ΔftsW
G
Cell volume (μm3)
****
****
***
IPTG
SH1000
ΔftsW
F
SH1000
ΔftsW
Frequency (%)
IPTG
No septum
Incomplete septum
Complete septum
Splitting cell
Multiple septa
Splitting without septum
Other

Supplementary figure 3. Essentiality of FtsW for growth, survival, cell size regulation and progression of the septal plate.

A. Genetic arrangement of the isogenic (SH1000) and the P*spac-ftsW* conditional lethal (Δ*ftsW*, SJF5761) strains. **B.** Growth (top) and viability (bottom) of SH1000 and Δ*ftsW* (SJF5761) cells in the presence and absence of IPTG. Means and standard deviations of three independent experiments are shown. **C.** Western blot of whole cell lysates of SH1000 and Δ*ftsW* grown for 1 h in the presence or absence of IPTG. Anti-FtsW antibodies were used for detection. Detection of YmdA with anti-YmdA antibodies is shown as loading control. **D.** Western blot signal quantification (based on image in C). Results are the average and standard deviation of three independent repeats. P values were determined by two-tailed unpaired t-test (***, $P = 0.0001$; **, $P = 0.0047$). **E.** Fluorescence microscopy images of SH1000 and Δ*ftsW* cells grown for 1 h with and without IPTG. PG was labelled by 30 min incubation with ADA-DA. Staining with NHS-ester shows cell morphology. Images are average z stack intensity projections. Scale bars, 1 µm. Images are representative of two independent experiments. **F.** Classification of cell division stages and septal defects based on ADA-DA labelling (based on images in E), $n = 300$ cells per sample. **G.** Cell volumes of SH1000 and Δ*ftsW* cells determined from images of cells stained with NHS-ester 555 (based on images in E). Each circle indicates a single cell. $n = 50$ cells per sample. P values were determined by Mann–Whitney U tests (****, $P < 0.0001$; ***, $P = 0.0007$).

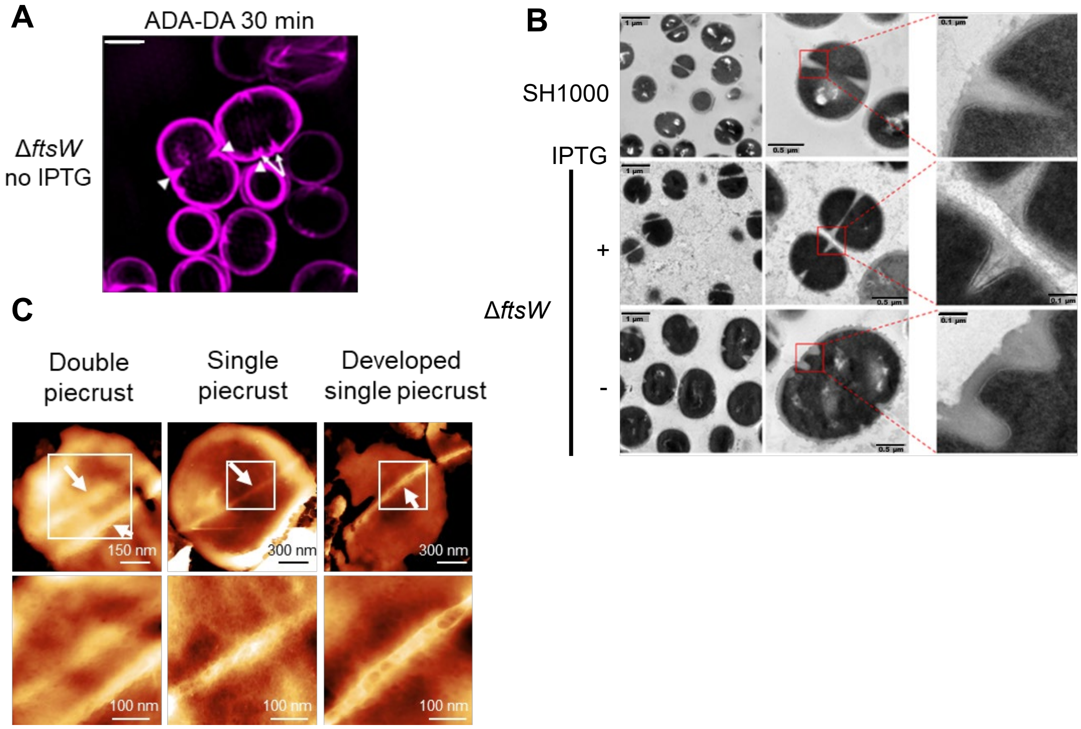

Supplementary figure 4. Septal defects in the absence of FtsW.

A. Structured Illumination Microscopy (SIM) images of Δ*ftsW* (SJF5761) cells grown for 1h without IPTG showing characteristic septal defects (white arrows). Images are average z stack intensity projections. Scale bars, 2 μm. Images are representative of two independent experiments. **B.** TEM of SH1000 and Δ*ftsW* grown for 1 h with or without IPTG. **C.** Atomic Force Microscopy (AFM) showing the piecrust features of Δ*ftsW* grown in the absence of IPTG. Double and single piecrust structures are shown (white arrows). For comparison with SH1000 and FtsL-depleted cells, see fig. 1F.

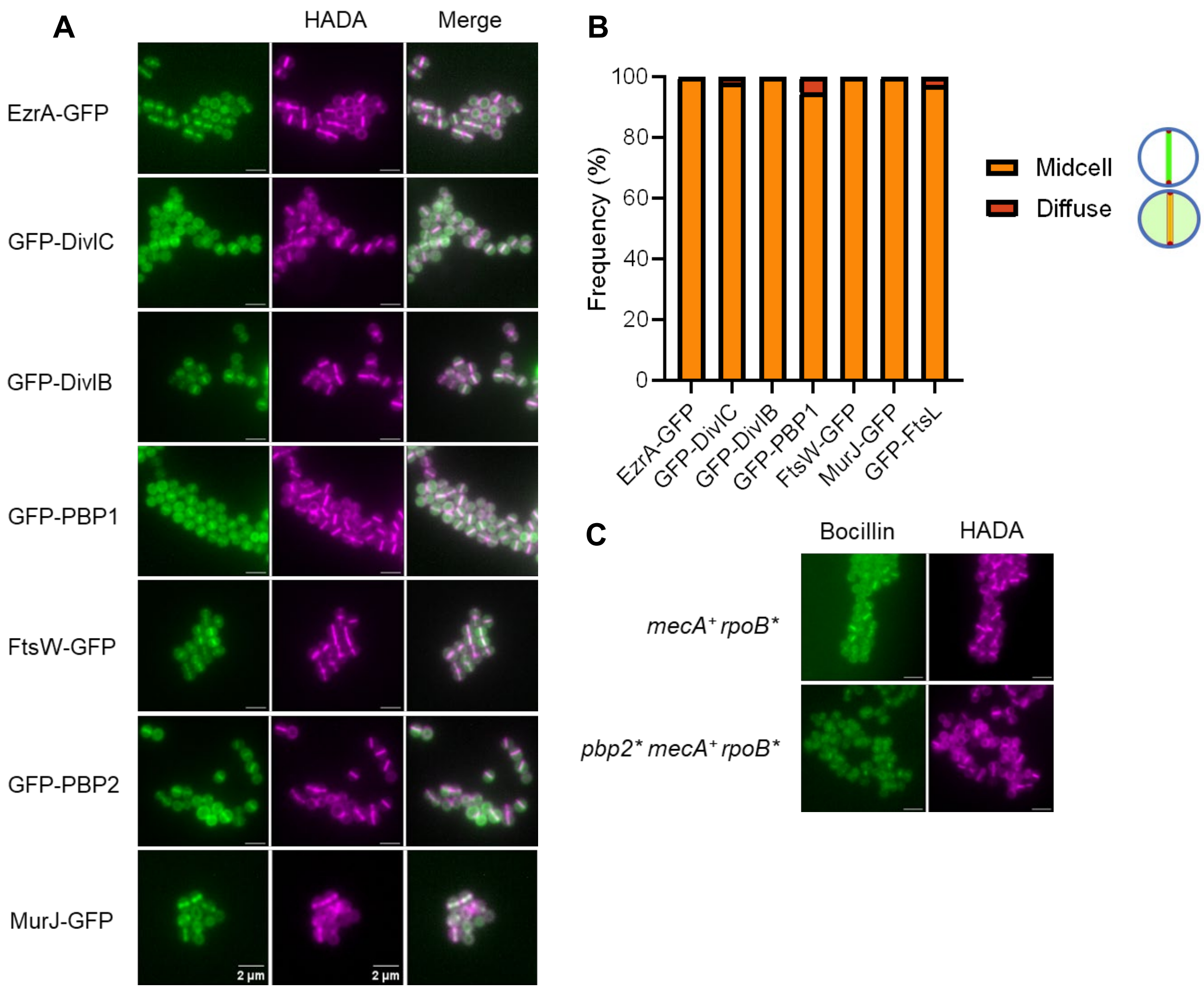

Supplementary figure 5. Mid-cell localisation of the division machinery in SH1000 during cell division and Bocillin binding in the absence of PBP2.

A. Fluorescence microscopy images of SH1000 cells expressing fluorescent fusions of *ftsL* (GFP-FtsL, SJF5929), *ezrA* (EzrA-GFP, VF104), *divIC* (GFP-DivIC, SJF5990), *divIB* (GFP-DivIB, SJF5573), *pbp1* (GFP-PBP1, SJF5927), *ftsW* (FtsW-GFP, SJF5768), *pbp2* (GFP-PBP2, SJF5541), and *murJ* (MurJ-GFP, SJF5965) grown for 1 h. PG was labelled by 30 min incubation with HADA. Images are average z stack intensity projections. Scale bars, 2 µm. Images are representative of two independent experiments. **B.** Quantification of fluorescent fusion localisation in SH1000 (based on images in a). Only cells showing incorporation of HADA at mid-cell were considered. n > 100 cells per sample. **C.**

Fluorescence microscopy images of SH1000 *mecA*⁺ *rpoB** (SJF5046) and *mecA*⁺ *rpoB** *pbp2** (SJF5809) grown for 2 h are shown. PG was labelled by 30 min incubation with HADA followed by the addition of Bocillin for 5 min. Images are average z stack intensity projections. Scale bars, 2 µm. Images are representative of two independent experiments.

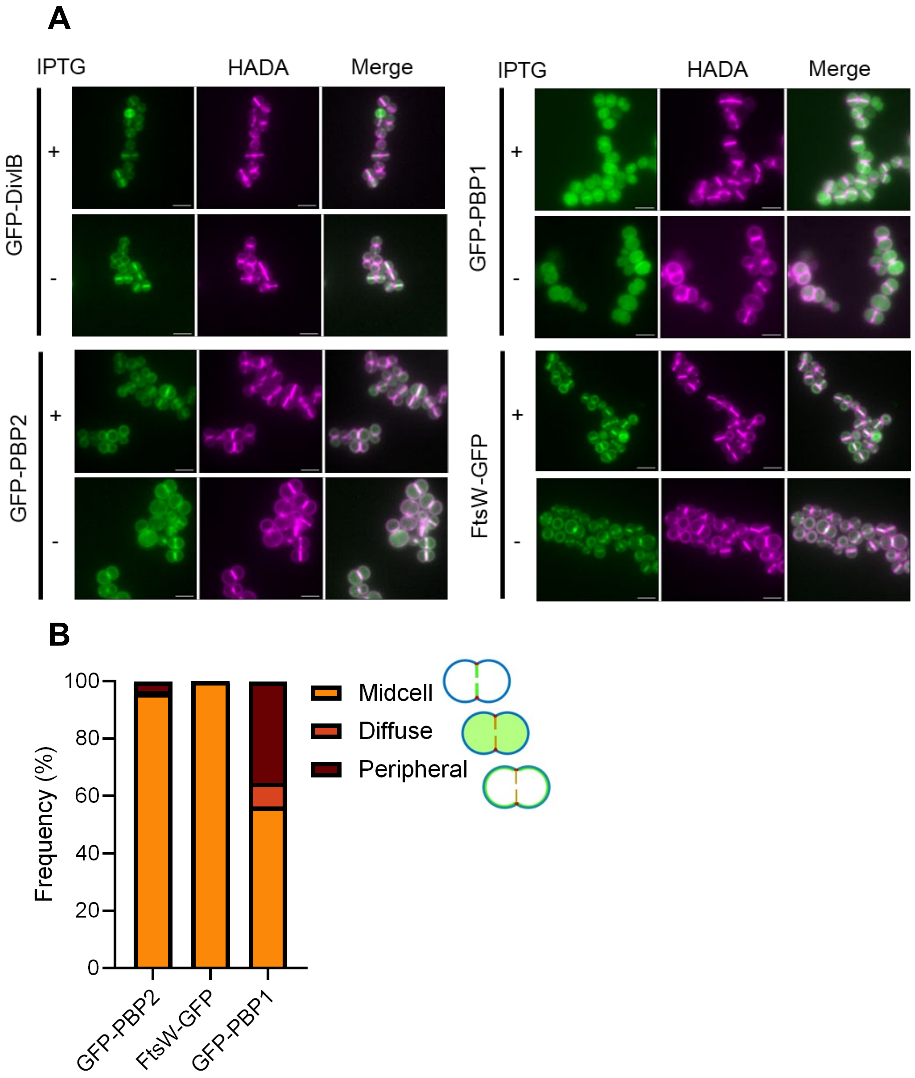

Supplementary figure 6. DivIB facilitates the localisation of PBP1 to mid-cell during septum development.

A. Fluorescence microscopy images of Δ*divIB* expressing fluorescent fusions of *divIB* (GFP-DivIB, SJF5701*), pbp1* (GFP-PBP1, SJF5930), *ftsW* (FtsW-GFP, SJF5770), and *pbp2* (GFP-PBP2, 5699SJF) grown for 2 h in the presence or absence of IPTG. PG was labelled by 30 min incubation with HADA. Images are average z stack intensity projections. Scale bars, 2 µm. Images are representative of two independent experiments. **B.** Quantification of fluorescent fusion localisation in Δ*divIB* in the absence of IPTG (based on images in A). Only cells showing incorporation of HADA at mid-cell were considered. *n* > 100 cells per sample.

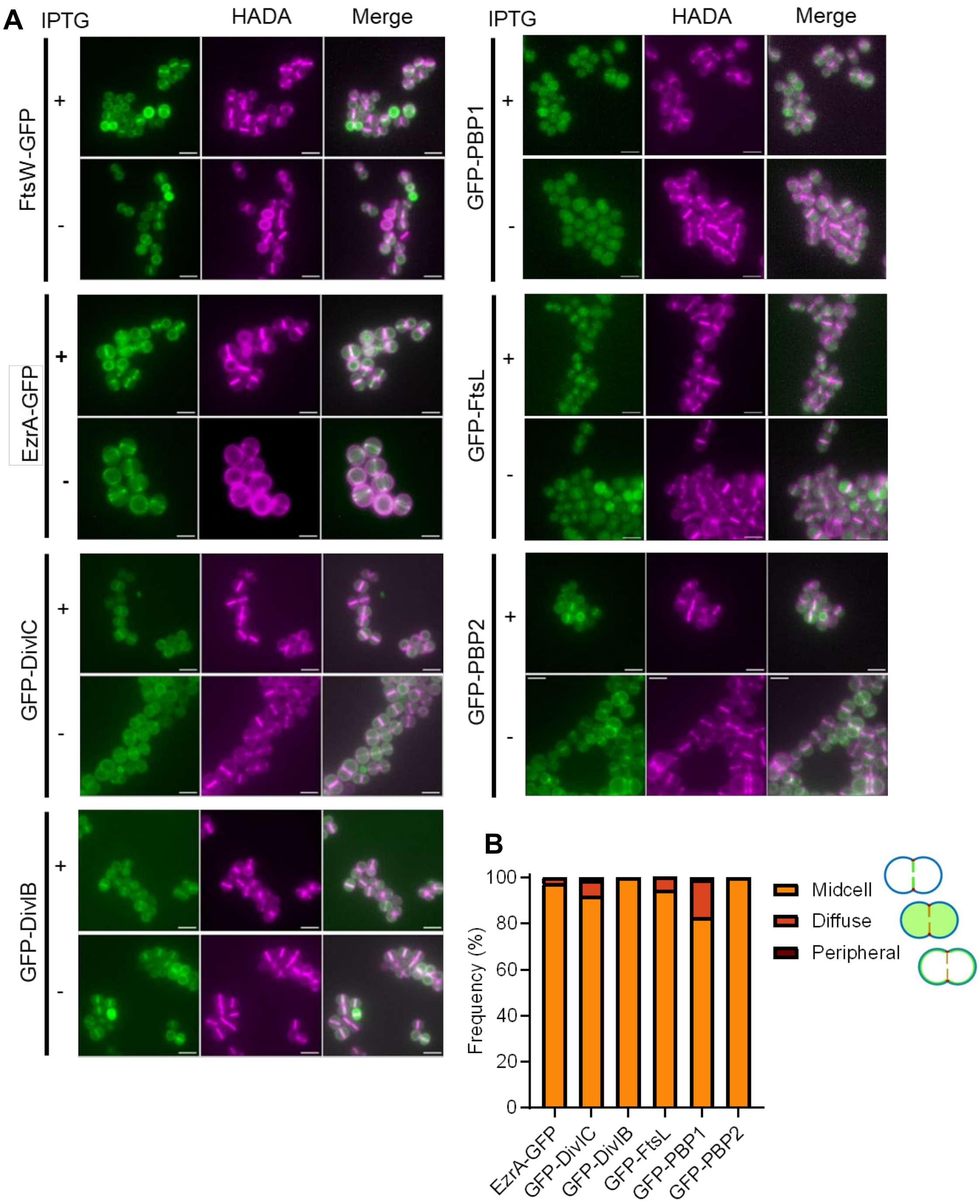

Supplementary figure 7. Localisation of division proteins to the cell division site is independent of FtsW.

A. Fluorescence microscopy images of SH1000 Δ*ftsW* expressing fluorescent fusions of *ftsW* (FtsW-GFP, SJF5946), *ezrA* (EzrA-GFP, SJF5945), *divIC* (GFP-DivIC, SJF5944), *divIB* (GFP-DivIB, SJF5943), *pbp1* (GFP-PBP1, SJF5947), *ftsL* (GFP-FtsL, SJF5948),

and pbp2 (GFP-PBP2, SJF5942). Cells were grown for 1 h in the presence or absence of IPTG. PG was labelled by 30 min incubation with HADA. Images are average z stack intensity projections. Scale bars, 2 µm. Images are representative of two independent experiments. **B.** Quantification of fluorescent fusion localisation in SH1000 (based on images in A). Only cells showing incorporation of HADA at mid-cell were considered. *n* > 100 per sample.

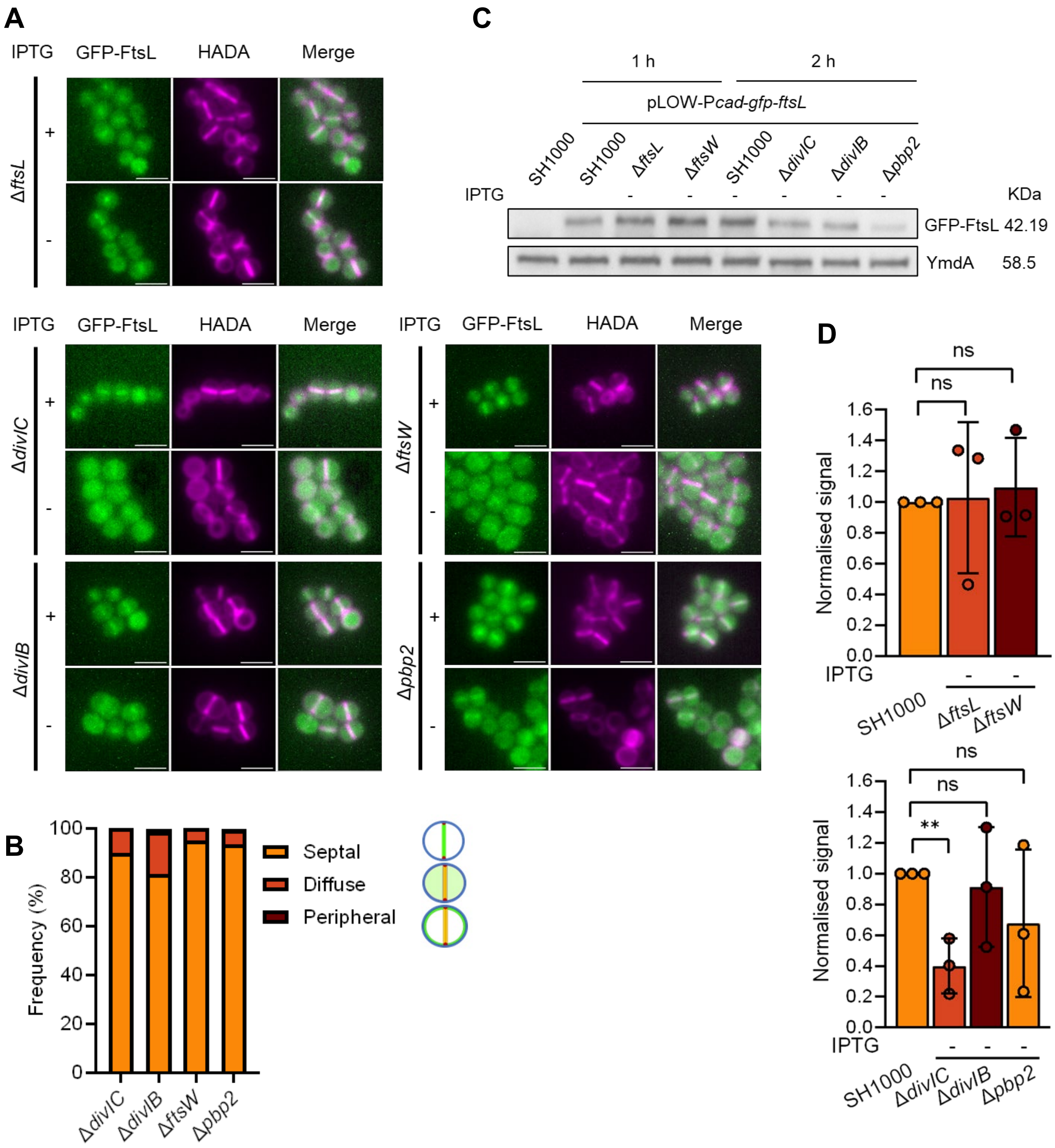

Supplementary figure 8. FtsL localises at mid-cell independently of the absence of other members of the divisome.

A. Fluorescence microscopy images of Δ*ftsL* (SJF5936), Δ*divIC* (SJF5935), Δ*divIB* (SJF5934), Δ*pbp2* (SJF6004), and Δ*ftsW* (SJF5948) expressing GFP-FtsL by the addition of 0.2 µm Cd were incubated in the presence or absence of IPTG for 1 (Δ*ftsL* and Δ*ftsW*) and 2 h (Δ*divIC*, Δ*divIB*, Δ*pbp2*). PG was labelled by 30 min incubation with HADA.

Images are average z stack intensity projections. Scale bars, 2 μm. Images are representative of two independent experiments. **B.** Quantification of GFP-FtsL localisation at mid-cell (based on images in A). Only cells showing incorporation of HADA at mid-cell were considered. *n* > 100 cells per sample. **C.** Western blot of whole cell lysates of SH1000 and the conditional lethal strains expressing GFP-FtsL by the addition of 2 μM $CdCl_2$ (as in A). Cells were grown for 1h (Δ*ftsL* and Δ*ftsW*) or 2 h (Δ*divIC*, Δ*divIB* and Δ*pbp2*) in the absence of IPTG. Anti-GFP antibodies were used for detection. Lysates of SH1000 were included as a negative control. YmdA blot with anti-YmdA antibodies is shown as loading control. **D.** Western blot signal quantification (based on images as in C). Results are the average and standard deviation of three independent repeats. *P* values were determined by two-tailed unpaired *t*-test (**, *P* = 0.0044).

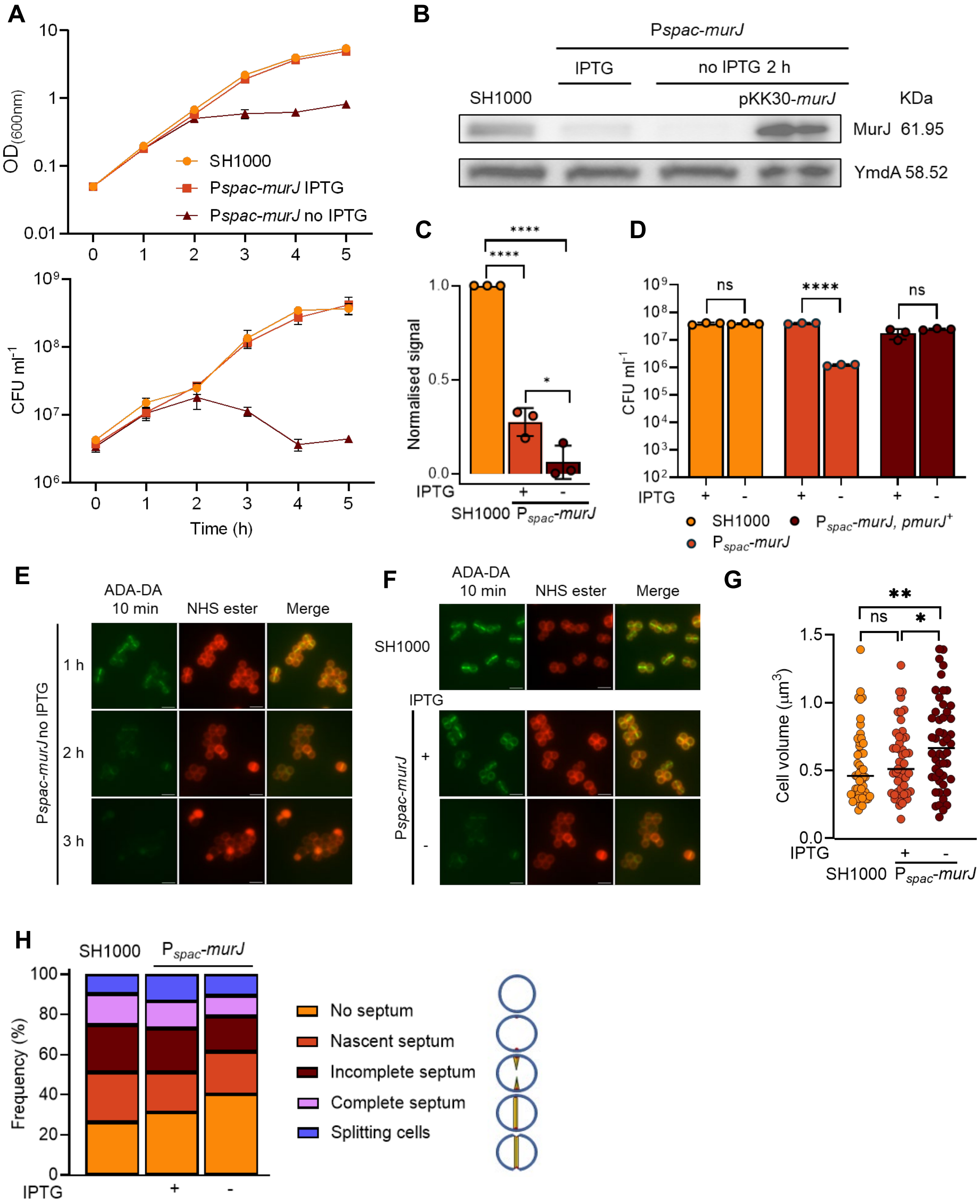

Supplementary figure 9. MurJ is essential for growth and survival.

A. Growth (top) and viability (bottom) of SH1000 and P$_{spac}$-*murJ* (SJF6065) strains in the presence and absence of IPTG. Mean and standard deviations of three independent

experiments are shown. **B.** Western blot of whole cell lysates of SH1000 and P_{spac}-*murJ* after 2 h incubation in the presence and absence of IPTG, a lysate of P_{spac}-*murJ* grown without IPTG and complemented with pKK30-*murJ* (SJF6071) is shown as a positive control. MurJ was detected using anti-MurJ antibodies. YmdA detection with anti-YmdA was included as loading control. **C.** Western blot signal quantification (based on images in B). Results are the average and standard deviation of three independent repeats. *P* values were determined by two-tailed unpaired *t*-test (****, $P = 0.0001$; *, $P = 0.0331$). **D.** Plating efficiency of SH1000 and P_{spac}-*murJ* cells grown for 2 h in the presence of IPTG followed by overnight incubation in solid medium with and without IPTG. Data is the mean and standard deviations of three independent experiments. The *P* value was determined by two-tailed unpaired *t*-test (****, $P < 0.0001$). **E.** Fluorescence microscopy images of P_{spac}-*murJ* cells grown for 1, 2 and 3 h without IPTG. PG synthesis was visualised by 10 min incubation with ADA-DA. Staining with NHS-ester shows cell morphology. Images are average z stack intensity projections. Scale bars, 2 µm. Images are representative of two independent experiments. **F.** Fluorescence microscopy images of SH1000 and P_{spac}-*murJ* cells grown for 2 with or without IPTG. PG synthesis was visualised by 10 min incubation with ADA-DA. Staining with NHS-ester shows cell morphology. Images are average z stack intensity projections. Scale bars, 2 µm. Images are representative of two independent experiments. **G.** Cell volumes of SH1000 and P_{spac}-*murJ* cells determined from images of cells stained with NHS ester 555 (based on images in F). Each circle indicates a single cell. $n = 50$ cells per sample. *P* values were determined by Mann–Whitney U tests (**, $P = 0.0068$; *, $P = 0.0487$). **H.** Classification of cellular division stages based on NHS ester 555 staining (based on images in F), $n = 300$ cells per sample.

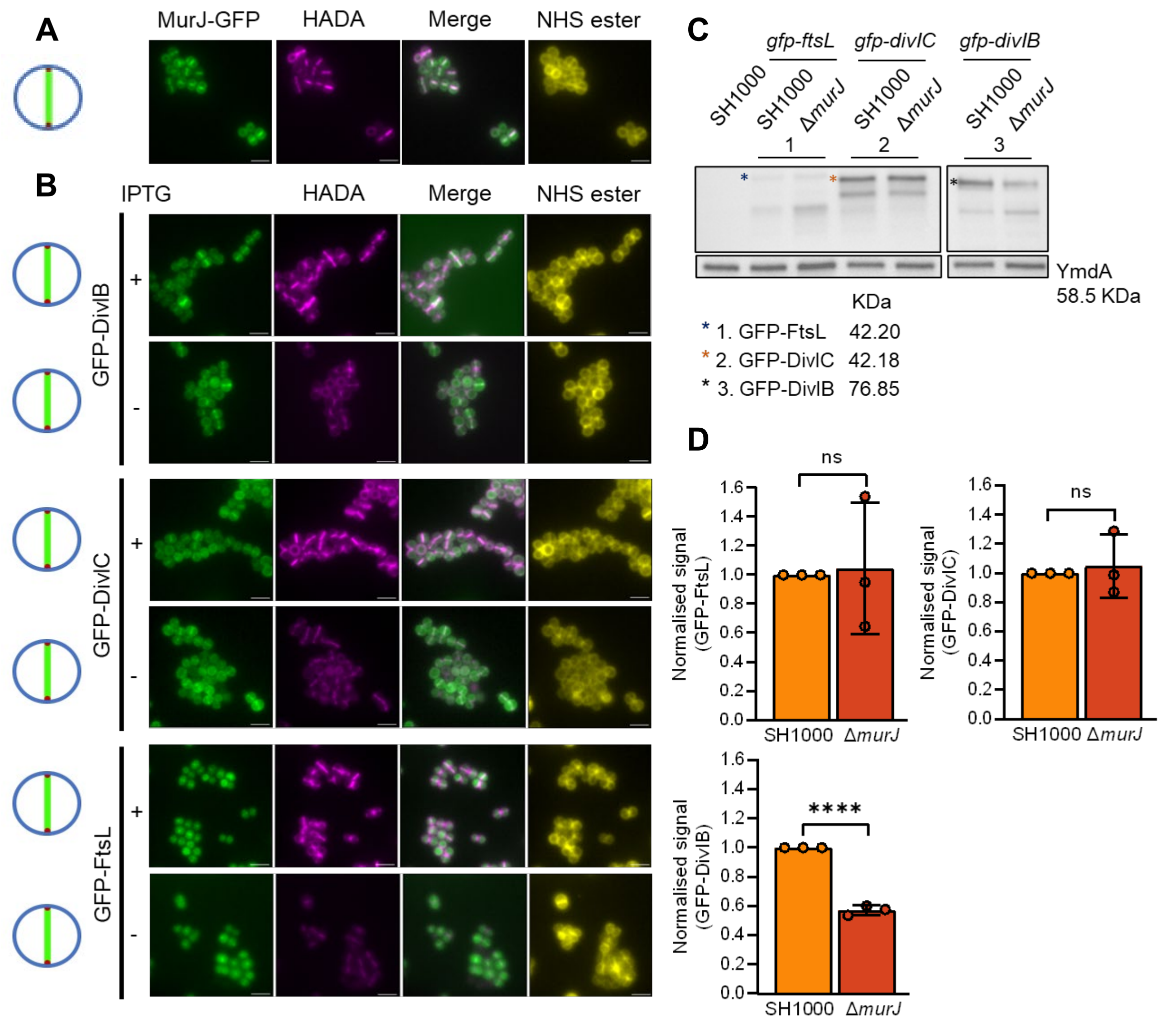

Supplementary figure 10. DivIB, DivIC and FtsL localise to the division site independently of MurJ.

A. Fluorescence microscopy images of SH1000 expressing a *murJ* fluorescent fusion (SJF5965) showing septal localisation. **B.** Fluorescence microscopy images of Δ*murJ* expressing fluorescent fusions of *divIB* (GFP-DivIB, SJF6081), *divIC* (GFP-DivIC, SJF6072), and *ftsL* (GFP-FtsL, SJF6082). Cells were grown for 2 h in the presence and absence of IPTG. PG synthesis was labelled by 5 min incubation with HADA. Staining with NHS ester 555 shows cell morphology Images are average z stack intensity projections. Scale bars, 2 µm. Images are representative of two independent experiments. **C.** Western blot of whole cell lysates of SH1000 and Δ*murJ* expressing GFP-FtsL, GFP-DivIC or GFP-DivIB after 2 h incubation in the presence and absence of

IPTG, a lysate of SH1000 was included as a negative control. Anti-GFP antibodies were used for detection. YmdA detection with anti-YmdA was included as loading control. Asterisks indicate the position of the protein fusion. Bands below show products of protein degradation. **D.** Western blot signal quantification (based on images as in C. Only full-length proteins indicated by asterisks next to band were included in the analysis). Results are the average and standard deviation of three independent repeats. *P* values were determined by two-tailed unpaired *t*-test (****, $P < 0.0001$).

Name	Relevant genotype/markers	Source
Escherichia coli		
NEB5a	*fhuA2 (argF-lacZ)U169 phoA glnV44 80 (lacZ)*M15 *gyrA96 recA1 relA1 endA1 thi-1 hsdR17*; Amp^R	NEB
Staphylococcus aureus		
SH1000	Functional *rsbU*$^+$ derivative of 8325–4	(78)
RN4220	Restriction deficient transformation recipient	(79)
CYL316	RN4220 pCL112Δ19; Cm^R	(80)
VF17	SH1000 pGL485 (*lacI*); Cm^R	(64)
SJF5450	SH1000 *geh*::P*spac-divIC*, *divIC*::*tet* (*lacI*); Kan^R, Tet^R, Cm^R	(30)
SJF3883	SH1000 *spa*::*kan*, *geh*::P*spac-divIB*, Δ*divIB* (*lacI*); Tet^R, Cm^R	(29)
SJF5665	SH1000 *geh*::P*spac-ftsL*, *ftsL*::*tet* (*lacI*); Kan^R, Tet^R, Cm^R	This study
SJF5666	SH1000 *geh*::P*spac-gfp-ftsL*, *ftsL*::*tet* (*lacI*); Kan^R, Tet^R, Cm^R	This study
SJF5761	SH1000 *geh*::P*spac-ftsW*, Δ*ftsW* (*lacI*); Tet^R, Cm^R	This study
SJF5630	SH1000 *geh*::P*spac-pbp2*, *pbp2*::*tet* (*lacI*); Kan^R, Tet^R, Cm^R	(11)
SJF6065	SH1000, *murJ::*pP*spac-murJ, (lacI)*; Ery/Lin^R, Cm^R	This study
SJF6071	SJF6065, pKK30-*murJ*, Ery/Lin^R, Cm^R, Tm^R	This study
SJF5696	SJF5665, *lysA*::*ezrA-gfp*; Kan^R, Tet^R, Cm^R, Ery/Lin^R	This study
SJF5693	SJF5665, pLOW-P*pcn-gfp-divIC*; Kan^R, Tet^R, Cm^R, Ery/Lin^R	This study
SJF5694	SJF5665, pLOW-P*pcn-gfp-divIB*; Kan^R, Tet^R, Cm^R, Ery/Lin^R	This study
SJF5932	SJF5665, pLOW-P*cad-gfp-pbp1*; Kan^R, Tet^R, Cm^R, Ery/Lin^R	This study
SJF5769	SJF5665, pLOW-P*pcn-ftsW-gfp*; Kan^R, Tet^R, Cm^R, Ery/Lin^R	This study
SJF5695	SJF5665, pLOW-P*pcn-gfp-pbp2*; Kan^R, Tet^R, Cm^R, Ery/Lin^R	This study
SJF5929	VF17, pLOW-P*cad-gfp-ftsL*; Cm^R, Ery/Lin^R	This study
VF104	VF17, *lysA::ezrA-gfp*; Cm^R, Ery/Lin^R	(64)
SJF5990	VF17, pLOW-P*pcn-gfp-divIC*; Cm^R, Ery/Lin^R	This study
SJF5573	VF17, pLOW-P*pcn-gfp-divIB*; Cm^R, Ery/Lin^R	(30)
SJF5927	VF17, pLOW-P*cad-gfp-pbp1*; Cm^R, Ery/Lin^R	This study
SJF5768	VF17, pLOW-P*pcn-ftsW-gfp*; Cm^R, Ery/Lin^R	This study
SJF5541	VF17, pLOW-P*pcn-gfp-pbp2*; Cm^R, Ery/Lin^R	(30)
SJF5046	*lysA::pmecA*, *rpoB*-H929Q; Kan^R	(34)
SJF5809	*lysA::pmecA*, *rpoB*-H929Q, *geh*::P*spac-pbp2**(TP-) *pbp2::tet*, (*lacI*); Kan^R, Tet^R, Cm^R, Ery/Lin^R	(11)
SJF5936	SJF5665, pLOW-P*cad-gfp-ftsL*; Kan^R, Tet^R, Cm^R, Ery/Lin^R	This study
SJF5935	SJF5450, pLOW-P*cad-gfp-ftsL*; Kan^R, Tet^R, Cm^R, Ery/Lin^R	This study
SJF5934	SJF3883, pLOW-P*cad-gfp-ftsL*; Kan^R, Tet^R, Cm^R, Ery/Lin^R	This study
SJF6004	SJF5630, pLOW-P*cad-gfp-ftsL*; Kan^R, Tet^R, Cm^R, Ery/Lin^R	This study
SJF5948	SJF5761, pLOW-P*cad-gfp-ftsL*; Kan^R, Tet^R, Cm^R, Ery/Lin^R	This study
SJF5701	SJF3883, pLOW-P*pcn-gfp-divIB*; Tet^R, Cm^R, Ery/Lin^R	This study
SJF5930	SJF3883, pLOW-P*cad-gfp-pbp1*; Tet^R, Cm^R, Ery/Lin^R	This study
SJF5770	SJF3883, pLOW-P*pcn-ftsW-gfp*; Tet^R, Cm^R, Ery/Lin^R	This study
SJF5699	SJF3883, pLOW-P*pcn-gfp-pbp2*; Tet^R, Cm^R, Ery/Lin^R	This study
SJF5946	SJF5761, pLOW-P*pcn-ftsW-gfp*; Kan^R, Tet^R, Cm^R, Ery/Lin^R	This study
SJF5945	SJF5761, *lysA::ezrA-gfp*; Kan^R, Tet^R, Cm^R, Ery/Lin^R	This study
SJF5944	SJF5761, pLOW-P*pcn-gfp-divIC*; Kan^R, Tet^R, Cm^R, Ery/Lin^R	This study
SJF5943	SJF5761, pLOW-P*pcn-gfp-divIB*; Kan^R, Tet^R, Cm^R, Ery/Lin^R	This study
SJF5947	SJF5761, pLOW-P*cad-gfp-pbp1*; Kan^R, Tet^R, Cm^R, Ery/Lin^R	This study
SJF5948	SJF5761, pLOW-P*cad-gfp-ftsL*; Kan^R, Tet^R, Cm^R, Ery/Lin^R	This study
SJF5942	SJF5761, pLOW-P*pcn-gfp-pbp2*; Kan^R, Tet^R, Cm^R, Ery/Lin^R	This study
SJF5781	SJF5665, pLOW-P*pcn-ftsL*; Kan^R, Tet^R, Cm^R, Ery/Lin^R	This study
SJF5782	SJF5665, pLOW-P*pcn-ftsL*ΔV124-N-133; Kan^R, Tet^R, Cm^R, Ery/Lin^R	This study
SJF5783	SJF5665, pLOW-P*pcn-ftsL*ΔK111-N133; Kan^R, Tet^R, Cm^R, Ery/Lin^R	This study
SJF5785	SJF5665, pLOW-P*pcn-ftsL*ΔA69-N133; Kan^R, Tet^R, Cm^R, Ery/Lin^R	This study
SJF5803	SJF5665, pLOW-P*pcn-ftsL*ΔA2-T41; Kan^R, Tet^R, Cm^R, Ery/Lin^R	This study
SJF5985	VF17, *pbp3::Tn*; Cm^R, Ery/Lin^R	This study
SJF5984	SJF5665, *pbp3::Tn*; Kan^R, Tet^R, Cm^R, Ery/Lin^R	This study
SJF5982	SJF3883, *pbp3::Tn*; Tet^R, Cm^R, Ery/Lin^R	This study
SJF6009	SJF5450, *pbp3::Tn*; Kan^R, Tet^R, Cm^R, Ery/Lin^R	This study

SJF5983	SJF5761, *pbp3::Tn*; Kan^R, Tet^R, Cm^R, Ery/Lin^R	This study
SJF5989	VF17, *pbp4::Tn*; Cm^R, Ery/Lin^R	This study
SJF5988	SJF5665, *pbp4::Tn*; Kan^R, Tet^R, Cm^R, Ery/Lin^R	This study
SJF5986	SJF3883, *pbp4::Tn*; Tet^R, Cm^R, Ery/Lin^R	This study
SJF6010	SJF5450, *pbp4::Tn*; Kan^R, Tet^R, Cm^R, Ery/Lin^R	This study
SJF5987	SJF5761, *pbp4::Tn*; Kan^R, Tet^R, Cm^R, Ery/Lin^R	This study
SJF5965	VF17, pLOW-P*pcn-murJ-gfp*; Cm^R, Ery/Lin^R	This study
SJF5957	SJF5665, pLOW-P*pcn-murJ-gfp*; Kan^R, Tet^R, Cm^R, Ery/Lin^R	This study
SJF5959	SJF5450, pLOW-P*pcn-murJ-gfp*; Kan^R, Tet^R, Cm^R, Ery/Lin^R	This study
SJF5960	SJF3883, pLOW-P*pcn-murJ-gfp*; Tet^R, Cm^R, Ery/Lin^R	This study
SJF6081	SJF6065, pLOW-P*pcn-gfp-divIB*; Ery/Lin^R, Cm^R, Kan^R	This study
SJF6072	SJF6065, pLOW-P*pcn-gfp-divIC*; Ery/Lin^R, Cm^R, Kan^R	This study
SJF6082	SJF6065, pLOW-P*cad-gfp-ftsL*; Ery/Lin^R, Cm^R, Kan^R	This study

Amp^R, ampicillin resistant; Ery^R, erythromycin resistant; Lin^R, lincomycin resistant; Tet^R, tetracycline resistant; Kan^R, kanamycin resistant; Cm^R, chloramphenicol resistant; Tm^R, trimethoprim resistant; -TP, deletion of the transpeptidase activity site.

Table S1. Strains

Name	Relevant genotype/markers	Source
pGL485	*E. coli* - *S. aureus* shuttle vector carrying a constitutively expressed *lacI* gene from *E. coli*; Spec^R, Cm^R	(81)
pKASBAR	pUC18 containing *attP* and a kanamycin resistance cassette; Amp^R, Kan^R	(29)
pOB-*tet*	pGEM3Zf(+) cloning vector containing the tetracycline resistance cassette from pAISH; Amp^R, Kan^R, Tet^R	(20)
pMUTIN	Vector containing a P*spac* promoter and an *E. coli* origin of replication. For insertional mutagenesis of targeted genes in the chromosome of *S. aureus*. Ery^R	(71)
pKK30	A highly stable vector for gene expression in *Staphylococcus* species; Tm^R	(82)
pMAD	*E. coli* - *S. aureus* shuttle vector with temperature-sensitive origin of replication in *S. aureus* and *bgaB* lacking promoter; Amp^R, Ery^R	(70)
pKASBAR-*ftsL*	pKASBAR-*kan*, P*spac-ftsL*; Amp^R, Kan^R	Genewiz UK Ltd
pKASBAR-*gfp-ftsL*	pKASBAR-*kan*, P*spac-gfp-ftsL*; Amp^R, Kan^R	Genewiz UK Ltd
pMAD-U-*tet*-D*ftsL*	pMAD carrying 964 bp upstream *ftsL* followed by a tetracycline resistance cassette and 1000 bp downstream from *ftsL* nucleotide 223 onwards; Amp^R, Ery^R, Tet^R	This study
pKASBAR-*ftsW*	pKASBAR-*tet*, P*spac-ftsW*; Amp^R, Tet^R	This study
pMAD-U-D*ftsW*	pMAD carrying 782 bp upstream and 777 bp downstream *ftsW*; Amp^R, Ery^R	This study
pMUTIN-HA-*murJ*	pMUTIN carrying *murJ* under control of a P*spac* promoter Ery^R	This study
pKK30-*murJ*	pKK30 carrying *murJ* under control of its own promoter; Tm^R	This study
pLOW	pSK41-type low copy number plasmid; Amp^R, Ery^R	(83)
pLOW-P*pcn-gfp-divIC*	pLOW carrying *gfp-divIC* under control of the penicillinase constitutive promoter (P*pcn*); Ery^R or Kan^R	(30)
pLOW-P*pcn-gfp-divIB*	pLOW carrying a *gfp-divIB* fusion under control of the penicillinase constitutive promoter (P*pcn*); Ery^R	(30)
pLOW-P*pcn-gfp-divIB* (Kan)	pLOW carrying a *gfp-divIB* fusion under control of the penicillinase constitutive promoter (P*pcn*); Kan^R	This study
pLOW-P*cad-gfp-ftsL*	pLOW carrying a *gfp-ftsL* fusion under control of the cadmium inducible promoter (P*cad-cadC*); Ery^R or Kan^R	This study
pLOW-P*cad-gfp-pbp1*	pLOW carrying a *gfp-pbp1* fusion under control of the cadmium inducible promoter (P*cad-cadC*); Ery^R	This study
pLOW-P*pcn-ftsW-gfp*	pLOW expressing a *ftsW-gfp* fusion under control of the penicillinase constitutive promoter (P*pcn*); Ery^R	(30)
pLOW-P*pcn-gfp-pbp2*	pLOW expressing a *gfp-pbp2* fusion under control of the penicillinase constitutive promoter (P*pcn*); Ery^R	(30)
pLOW-P*pcn-murJ-gfp*	pLOW expressing a *murJ-gfp* fusion under control of the penicillinase constitutive promoter (P*pcn*); Ery^R	Genewiz UK Ltd
pLOW-P*pcn-ftsL*	pLOW carrying *ftsL* under control of the penicillinase constitutive promoter (P*pcn*); Ery^R	Genewiz UK Ltd
pLOW-P*pcn-ftsL*ΔV124-N-133	pLOW carrying a truncated *ftsL* gene under control of the penicillinase constitutive promoter (P*pcn*); Ery^R	Genewiz UK Ltd
pLOW-P*pcn-ftsL*ΔK111-N133	pLOW carrying a truncated *ftsL* gene under control of the penicillinase constitutive promoter (P*pcn*); Ery^R	Genewiz UK Ltd
pLOW-P*pcn-ftsL*ΔA69-N133	pLOW carrying a truncated *ftsL* gene under control of the penicillinase constitutive promoter (P*pcn*); Ery^R	Genewiz UK Ltd
pLOW-P*pcn-ftsL*ΔA2-T41	pLOW carrying a truncated *ftsL* gene under control of the penicillinase constitutive promoter (P*pcn*); Ery^R	Genewiz UK Ltd

Spec^R, spectinomycin; Amp^R, ampicillin resistant; Ery^R, erythromycin resistant; Tet^R, tetracycline resistant; Kan^R, kanamycin resistant; Cm^R, chloramphenicol resistant; trimethoprim, Tm^R.

Table S2. Plasmids

**Supplementary table 3. Oligonucleotides used for the construction of the conditional lethal strains.**		
**Number**	**Name**	**Sequence 5' to 3'**
109	U-*ftsL* Fw	CGTTACACATTAACTAGACAAATGGAGGAATTGAAGTG
110	U-*ftsL* Rv	CTCCTAATTTTATAAATTGCTCCTTATTTAAGTATTTC
111	*tet* Fw	GCAATTTATAAAATTAGGAGGAGAAGCATC
112	*tet* Rv	CTGCAATCTTTCTCTCCCAAAGTTGATC
113	D-*ftsL* Fw	TTGGGAGAGAAAGATTGCAGATTTAGATTATAAAATAG
114	D-*ftsL* Rv	CCTCGCGTCGGGCGATATCGAATTTCTCCAGTTTTGGC
215	P*spac* Fw	CCTTTTTTTGCCCCGGGATCCGCAAAAAGTTGTTGACTTTATC
216	P*spac* Rv	TTGGCTAGTATTTTTTTAATATTCAGTCATCCAATTC
217	*ftsW* Fw	AACAATTAAGCTTGATATCGATCATTTGAAGTATAAATTGTAGAATTG
218	*ftsW* Rv	CAGCTATGACCATGATTACGTTAATTAAATTGTCTTCTTATATCAACTTTTTG
219	U-*ftsW* Fw	CCATGGTACCCGGGAGCTCGGTCGTACTATCTAATGTTATAGG
220	U-*ftsW* Rv	TTTTTTTAATATATTCAGTCATCCAATTCTAC
221	D-*ftsW* Fw	TGACTGAATATATTAAAAAAATACTAGCCAATATTTAGTAC
222	D*ftsW* Rv	CCTCGCGTCGGGCGATATCGTGCTACATCAATGATACG
785F	*murJ* HIndIII	AGGAAGCTTAAACATGAGATAGGGAGATTCGTAAT
786R	*murJ*_KpnI	ACAGGTACCTGTCGCTAATACAGGAATAAAGATAACAACCA
916	*murJ* Fw	AGTGACAGGCGATGCGGCCGCTAGCTACGCTTCCTCCAAATTTGTTACT
917	*murJ* Rv	TACTTTTGCTTGTAATTCATGATTCGGATCCTCATCGTAAAAACCTAACTCT

Table S3. Oligonucleotides

REFERENCES AND NOTES

1. A. Typas, M. Banzhaf, C. A. Gross, W. Vollmer, From the regulation of peptidoglycan synthesis to bacterial growth and morphology. *Nat. Rev. Microbiol.* **10**, 123–136 (2011).

2. M. G. Pinho, M. Kjos, J.-W. Veening, How to get (a)round: Mechanisms controlling growth and division of coccoid bacteria. *Nat. Rev. Microbiol.* **11**, 601–614 (2013).

3. C. R. Mahone, E. D. Goley, Bacterial cell division at a glance. *J. Cell Sci.* **133**, jcs237057 (2020).

4. N. Buddelmeijer, J. Beckwith, A complex of the *Escherichia coli* cell division proteins FtsL, FtsB and FtsQ forms independently of its localization to the septal region. *Mol. Microbiol.* **52**, 1315–1327 (2004).

5. M. Noirclerc-Savoye, A. Le Gouëllec, C. Morlot, O. Dideberg, T. Vernet, A. Zapun, *In vitro* reconstitution of a trimeric complex of DivIB, DivIC and FtsL, and their transient co-localization at the division site in *Streptococcus pneumoniae*. *Mol. Microbiol.* **55**, 413–424 (2005).

6. R. A. Daniel, M. F. Noirot-Gros, P. Noirot, J. Errington, Multiple interactions between the transmembrane division proteins of *Bacillus subtilis* and the role of FtsL instability in divisome assembly. *J. Bacteriol.* **188**, 7396–7404 (2006).

7. R. D. Turner, W. Vollmer, S. J. Foster, Different walls for rods and balls: The diversity of peptidoglycan. *Mol. Microbiol.* **91**, 862–874 (2014).

8. W. Vollmer, S. J. Seligman, Architecture of peptidoglycan: More data and more models. *Trends Microbiol.* **18**, 59–66 (2010).

9. E. R. Rojas, G. Billings, P. D. Odermatt, G. K. Auer, L. Zhu, A. Miguel, F. Chang, D. B. Weibel, J. A. Theriot, K. C. Huang, The outer membrane is an essential load-bearing element in Gram-negative bacteria. *Nature* **559**, 617–621 (2018).

10. V. R. Matias, T. J. Beveridge, Native cell wall organization shown by cryo-electron microscopy confirms the existence of a periplasmic space in *Staphylococcus aureus*. *J. Bacteriol.* **188**, 1011–1021 (2006).

11. A. F. Adedeji-Olulana, K. Wacnik, L. Lafage, L. Pasquina-Lemonche, M. Tinajero-Trejo, J. A. F. Sutton, B. Bilyk, S. E. Irving, C. J. Portman Ross, O. J. Meacock, S. A. Randerson, E. Beattie, D. S. Owen, J. Florence, W. M. Durham, D. P. Hornby, R. M. Corrigan, J. Green, J. K. Hobbs, S. J. Foster, Two codependent routes lead to high-level MRSA. *Science* **386**, 573–580 (2024).

12. T. J. Silhavy, D. Kahne, S. Walker, The bacterial cell envelope. *Cold Spring Harb. Perspect. Biol.* **2**, a000414 (2010).

13. M. L. Atilano, P. M. Pereira, J. Yates, P. Reed, H. Veiga, M. G. Pinho, S. R. Filipe, Teichoic acids are temporal and spatial regulators of peptidoglycan cross-linking in *Staphylococcus aureus*. *Proc. Natl. Acad. Sci. U.S.A.* **107**, 18991–18996 (2010).

14. G. Xia, T. Kohler, A. Peschel, The wall teichoic acid and lipoteichoic acid polymers of *Staphylococcus aureus*. *Int. J. Med. Microbiol.* **300**, 148–154 (2010).

15. X. Yang, Z. Lyu, A. Miguel, R. McQuillen, K. C. Huang, J. Xiao, GTPase activity-coupled treadmilling of the bacterial tubulin FtsZ organizes septal cell wall synthesis. *Science* **355**, 744–747 (2017).

16. A. W. Bisson-Filho, Y.-P. Hsu, G. R. Squyres, E. Kuru, F. Wu, C. Jukes, Y. Sun, C. Dekker, S. Holden, M. S. VanNieuwenhze, Y. V. Brun, E. C. Garner, Treadmilling by FtsZ filaments drives peptidoglycan synthesis and bacterial cell division. *Science* **355**, 739–743 (2017).

17. J. M. Monteiro, A. R. Pereira, N. T. Reichmann, B. M. Saraiva, P. B. Fernandes, H. Veiga, A. C. Tavares, M. Santos, M. T. Ferreira, V. Macário, M. S. VanNieuwenhze, S. R. Filipe, M. G. Pinho, Peptidoglycan synthesis drives an FtsZ-treadmilling-independent step of cytokinesis. *Nature* **554**, 528–532 (2018).

18. R. D. Turner, E. C. Ratcliffe, R. Wheeler, R. Golestanian, J. K. Hobbs, S. J. Foster, Peptidoglycan architecture can specify division planes in *Staphylococcus aureus*. *Nat. Commun.* **1**, 26 (2010).

19. L. Pasquina-Lemonche, J. Burns, R. D. Turner, S. Kumar, R. Tank, N. Mullin, J. S. Wilson, B. Chakrabarti, P. A. Bullough, S. J. Foster, J. K. Hobbs, The architecture of the Gram-positive bacterial cell wall. *Nature* **582**, 294–297 (2020).

20. V. A. Lund, K. Wacnik, R. D. Turner, B. E. Cotterell, C. G. Walther, S. J. Fenn, F. Grein, A. J. Wollman, M. C. Leake, N. Olivier, A. Cadby, S. Mesnage, S. Jones, S. J. Foster, Molecular coordination of *Staphylococcus aureus* cell division. *eLife* **7**, e32057 (2018).

21. M. Wang, G. Buist, J. M. van Dijl, *Staphylococcus aureus* cell wall maintenance—The multifaceted roles of peptidoglycan hydrolases in bacterial growth, fitness, and virulence. *FEMS Microbiol. Rev.* **46**, fuac025 (2022).

22. R. D. Turner, J. K. Hobbs, S. J. Foster, Atomic force microscopy analysis of bacterial cell wall peptidoglycan architecture. *Methods Mol. Biol.* **1440**, 3–9 (2016).

23. A. C. Y. Kuk, A. Hao, S.-Y. Lee, Structure and mechanism of the lipid flippase MurJ. *Annu. Rev. Biochem.* **91**, 705–729 (2022).

24. N. T. Reichmann, A. C. Tavares, B. M. Saraiva, A. Jousselin, P. Reed, A. R. Pereira, J. M. Monteiro, R. G. Sobral, M. S. VanNieuwenhze, F. Fernandes, M. G. Pinho, SEDS-bPBP pairs direct lateral and septal peptidoglycan synthesis in *Staphylococcus aureus*. *Nat. Microbiol.* **4**, 1368–1377 (2019).

25. P. Loskill, P. M. Pereira, P. Jung, M. Bischoff, M. Herrmann, M. G. Pinho, K. Jacobs, Reduction of the peptidoglycan crosslinking causes a decrease in stiffness of the *Staphylococcus aureus* cell envelope. *Biophys. J.* **107**, 1082–1089 (2014).

26. V. Srisuknimit, Y. Qiao, K. Schaefer, D. Kahne, S. Walker, Peptidoglycan cross-linking preferences of *Staphylococcus aureus* penicillin-binding proteins have implications for treating MRSA Infections. *J. Am. Chem. Soc.* **139**, 9791–9794 (2017).

27. K. Wacnik, V. A. Rao, X. Chen, L. Lafage, M. Pazos, S. Booth, W. Vollmer, J. K. Hobbs, R. J. Lewis, S. J. Foster, Penicillin-binding protein 1 (PBP1) of *Staphylococcus aureus* has multiple essential functions in cell division. *mBio* **13**, e0066922 (2022).

28. B. Salamaga, L. Kong, L. Pasquina-Lemonche, L. Lafage, M. von Und Zur Muhlen, J. F. Gibson, D. Grybchuk, A. K. Tooke, V. Panchal, E. J. Culp, E. Tatham, M. E. O'Kane, T. E. Catley, S. A. Renshaw, G. D. Wright, P. Plevka, P. A. Bullough, A. Han, J. K. Hobbs, S. J. Foster, Demonstration of the role of cell wall homeostasis in *Staphylococcus aureus* growth and the action of bactericidal antibiotics. *Proc. Natl. Acad. Sci. U.S.A.* **118**, e2106022118 (2021).

29. A. L. Bottomley, A. F. Kabli, A. F. Hurd, R. D. Turner, J. Garcia-Lara, S. J. Foster, *Staphylococcus aureus* DivIB is a peptidoglycan-binding protein that is required for a morphological checkpoint in cell division. *Mol. Microbiol.* **94**, 1041–1064 (2014).

30. M. Tinajero-Trejo, O. Carnell, A. F. Kabli, L. Pasquina-Lemonche, L. Lafage, A. Han, J. K. Hobbs, S. J. Foster, The *Staphylococcus aureus* cell division protein, DivIC, interacts with the cell wall and controls its biosynthesis. *Commun. Biol.* **5**, 1228 (2022).

31. R. R. Chaudhuri, A. G. Allen, P. J. Owen, G. Shalom, K. Stone, M. Harrison, T. A. Burgis, M. Lockyer, J. Garcia-Lara, S. J. Foster, S. J. Pleasance, S. E. Peters, D. J. Maskell, I. G. Charles, Comprehensive identification of essential *Staphylococcus aureus* genes using Transposon-Mediated Differential Hybridisation (TMDH). *BMC Genomics* **10**, 291 (2009).

32. E. Kuru, H. V. Hughes, P. J. Brown, E. Hall, S. Tekkam, F. Cava, M. A. de Pedro, Y. V. Brun, M. S. VanNieuwenhze, *In situ* probing of newly synthesized peptidoglycan in live bacteria with fluorescent D-amino acids. *Angew. Chem. Int. Ed. Engl.* **51**, 12519–12523 (2012).

33. J. M. Monteiro, P. B. Fernandes, F. Vaz, A. R. Pereira, A. C. Tavares, M. T. Ferreira, P. M. Pereira, H. Veiga, E. Kuru, M. S. VanNieuwenhze, Y. V. Brun, S. R. Filipe, M. G. Pinho, Cell shape dynamics during the staphylococcal cell cycle. *Nat. Commun.* **6**, 8055 (2015).

34. V. V. Panchal, C. Griffiths, H. Mosaei, B. Bilyk, J. A. F. Sutton, O. T. Carnell, D. P. Hornby, J. Green, J. K. Hobbs, W. L. Kelley, N. Zenkin, S. J. Foster, Evolving MRSA: High-level

β-lactam resistance in *Staphylococcus aureus* is associated with RNA Polymerase alterations and fine tuning of gene expression. *PLOS Pathog.* **16**, e1008672 (2020).

35. P. Reed, M. L. Atilano, R. Alves, E. Hoiczyk, X. Sher, N. T. Reichmann, P. M. Pereira, T. Roemer, S. R. Filipe, J. B. Pereira-Leal, P. Ligoxygakis, M. G. Pinho, *Staphylococcus aureus* survives with a minimal peptidoglycan synthesis machine but sacrifices virulence and antibiotic resistance. *PLOS Pathog.* **11**, e1004891 (2015).

36. R. A. Daniel, E. J. Harry, V. L. Katis, R. G. Wake, J. Errington, Characterization of the essential cell division gene *ftsL*(*yIID*) of *Bacillus subtilis* and its role in the assembly of the division apparatus. *Mol. Microbiol.* **29**, 593–604 (1998).

37. J. A. F. Sutton, M. Cooke, M. Tinajero-Trejo, K. Wacnik, B. Salamaga, C. Portman-Ross, V. A. Lund, J. K. Hobbs, S. J. Foster, The roles of GpsB and DivIVA in *Staphylococcus aureus* growth and division. *Front. Microbiol.* **14**, 1241249 (2023).

38. A. Lupas, Coiled coils: New structures and new functions. *Trends Biochem. Sci.* **21**, 375–382 (1996).

39. A. Lupas, M. Van Dyke, J. Stock, Predicting coiled coils from protein sequences. *Science* **252**, 1162–1164 (1991).

40. S. Schäper, A. D. Brito, B. M. Saraiva, G. R. Squyres, M. J. Holmes, E. C. Garner, Z. Hensel, R. Henriques, M. G. Pinho, Cell constriction requires processive septal peptidoglycan synthase movement independent of FtsZ treadmilling in *Staphylococcus aureus*. *Nat. Microbiol.* **4**, 1049–1063 (2024).

41. A. W. Wyke, J. B. Ward, M. V. Hayes, N. A. Curtis, A role *in vivo* for penicillin-binding protein-4 of *Staphylococcus aureus*. *Eur. J. Biochem.* **119**, 389–393 (1981).

42. J. Huber, R. G. Donald, S. H. Lee, L. W. Jarantow, M. J. Salvatore, X. Meng, R. Painter, R. H. Onishi, J. Occi, K. Dorso, K. Young, Y. W. Park, S. Skwish, M. J. Szymonifka, T. S. Waddell, L. Miesel, J. W. Phillips, T. Roemer, Chemical genetic identification of

peptidoglycan inhibitors potentiating carbapenem activity against methicillin-resistant *Staphylococcus aureus*. *Chem. Biol.* **16**, 837–848 (2009).

43. J. Errington, L. J. Wu, Cell cycle machinery in *Bacillus subtilis*. *Subcell. Biochem.* **84**, 67–101 (2017).

44. S. Du, J. Lutkenhaus, Assembly and activation of the *Escherichia coli* divisome. *Mol. Microbiol.* **105**, 177–187 (2017).

45. L. M. Guzman, J. J. Barondess, J. Beckwith, FtsL, an essential cytoplasmic membrane protein involved in cell division in *Escherichia coli*. *J. Bacteriol.* **174**, 7716–7728 (1992).

46. J. M. Ghigo, D. S. Weiss, J. C. Chen, J. C. Yarrow, J. Beckwith, Localization of FtsL to the *Escherichia coli* septal ring. *Mol. Microbiol.* **31**, 725–737 (1999).

47. S. Masson, T. Kern, A. Le Gouellec, C. Giustini, J. P. Simorre, P. Callow, T. Vernet, F. Gabel, A. Zapun, Central domain of DivIB caps the C-terminal regions of the FtsL/DivIC coiled-coil rod. *J. Biol. Chem.* **284**, 27687–27700 (2009).

48. H. B. van den Berg van Saparoea, M. Glas, I. G. Vernooij, W. Bitter, T. den Blaauwen, J. Luirink, Fine-mapping the contact sites of the *Escherichia coli* cell division proteins FtsB and FtsL on the FtsQ protein. *J. Biol. Chem.* **288**, 24340–24350 (2013).

49. R. A. Daniel, J. Errington, Intrinsic instability of the essential cell division protein FtsL of *Bacillus subtilis* and a role for DivIB protein in FtsL turnover. *Mol. Microbiol.* **36**, 278–289 (2000).

50. M. Bramkamp, L. Weston, R. A. Daniel, J. Errington, Regulated intramembrane proteolysis of FtsL protein and the control of cell division in *Bacillus subtilis*. *Mol. Microbiol.* **62**, 580–591 (2006).

51. J. C. Chen, D. S. Weiss, J. M. Ghigo, J. Beckwith, Septal localization of FtsQ, an essential cell division protein in *Escherichia coli*. *J. Bacteriol.* **181**, 521–530 (1999).

52. D. S. Weiss, J. C. Chen, J. M. Ghigo, D. Boyd, J. Beckwith, Localization of FtsI (PBP3) to the septal ring requires its membrane anchor, the Z ring, FtsA, FtsQ, and FtsL. *J. Bacteriol.* **181**, 508–520 (1999).

53. N. Buddelmeijer, J. Beckwith, Assembly of cell division proteins at the *E. coli* cell center. *Curr. Opin. Microbiol.* **5**, 553–557 (2002).

54. S. L. Rowland, K. D. Wadsworth, S. A. Robson, C. Robichon, J. Beckwith, G. F. King, Evidence from artificial septal targeting and site-directed mutagenesis that residues in the extracytoplasmic beta domain of DivIB mediate its interaction with the divisomal transpeptidase PBP 2B. *J. Bacteriol.* **192**, 6116–6125 (2010).

55. J. Sievers, J. Errington, Analysis of the essential cell division gene *ftsL* of *Bacillus subtilis* by mutagenesis and heterologous complementation. *J. Bacteriol.* **182**, 5572–5579 (2000).

56. L. M. Guzman, D. S. Weiss, J. Beckwith, Domain-swapping analysis of FtsI, FtsL, and FtsQ, bitopic membrane proteins essential for cell division in *Escherichia coli*. *J. Bacteriol.* **179**, 5094–5103 (1997).

57. J. M. Ghigo, J. Beckwith, Cell division in *Escherichia coli*: Role of FtsL domains in septal localization, function, and oligomerization. *J. Bacteriol.* **182**, 116–129 (2000).

58. V. L. Katis, R. G. Wake, Membrane-bound division proteins DivIB and DivIC of *Bacillus subtilis* function solely through their external domains in both vegetative and sporulation division. *J. Bacteriol.* **181**, 2710–2718 (1999).

59. Y. Kawai, N. Ogasawara, *Bacillus subtilis* EzrA and FtsL synergistically regulate FtsZ ring dynamics during cell division. *Microbiology* **152**, 1129–1141 (2006).

60. M.-J. Tsang, T. G. Bernhardt, A role for the FtsQLB complex in cytokinetic ring activation revealed by an *ftsL* allele that accelerates division. *Mol. Microbiol.* **95**, 925–944 (2015).

61. K.-T. Park, S. Du, J. Lutkenhaus, Essential role for FtsL in activation of septal peptidoglycan synthesis. *mBio* **11**, e03012–e03020 (2020).

62. J. Fujita, Y. Maeda, C. Nagao, Y. Tsuchiya, Y. Miyazaki, M. Hirose, E. Mizohata, Y. Matsumoto, T. Inoue, K. Mizuguchi, H. Matsumura, Crystal structure of FtsA from *Staphylococcus aureus*. *FEBS Lett.* **588**, 1879–1885 (2014).

63. J. W. Graham, M. G. Lei, C. Y. Lee, Trapping and identification of cellular substrates of the *Staphylococcus aureus* ClpC chaperone. *J. Bacteriol.* **195**, 4506–4516 (2013).

64. V. R. Steele, A. L. Bottomley, J. Garcia-Lara, J. Kasturiarachchi, S. J. Foster, Multiple essential roles for EzrA in cell division of *Staphylococcus aureus*. *Mol. Microbiol.* **80**, 542–555 (2011).

65. M. G. Pinho, S. R. Filipe, H. de Lencastre, A. Tomasz, Complementation of the essential peptidoglycan transpeptidase function of penicillin-binding protein 2 (PBP2) by the drug resistance protein PBP2A in *Staphylococcus aureus*. *J. Bacteriol.* **183**, 6525–6531 (2001).

66. M. G. Pinho, J. Errington, Recruitment of penicillin-binding protein PBP2 to the division site of *Staphylococcus aureus* is dependent on its transpeptidation substrates. *Mol. Microbiol.* **55**, 799–807 (2005).

67. J. Sambrook, D. W. Russell, *Molecular Cloning: A Laboratory Manual* (CSHL Press, ed. 3, 2001), vol. 1.

68. D. G. Gibson, L. Young, R. Y. Chuang, J. C. Venter, C. A. Hutchison III, H. O. Smith, Enzymatic assembly of DNA molecules up to several hundred kilobases. *Nat. Methods* **6**, 343–345 (2009).

69. R. P. Novick, S. I. Morse, *In vivo* transmission of drug resistance factors between strains of *Staphylococcus aureus*. *J. Exp. Med.* **125**, 45–59 (1967).

70. M. Arnaud, A. Chastanet, M. Débarbouillé, New vector for efficient allelic replacement in naturally nontransformable, low-GC-content, gram-positive bacteria. *Appl. Environ. Microbiol.* **70**, 6887–6891 (2004).

71. V. Vagner, E. Dervyn, S. D. Ehrlich, A vector for systematic gene inactivation in *Bacillus subtilis*. *Microbiology* **144**, 3097–3104 (1998).

72. E. Charpentier, A. I. Anton, P. Barry, B. Alfonso, Y. Fang, R. P. Novick, Novel cassette-based shuttle vector system for gram-positive bacteria. *Appl. Environ. Microbiol.* **70**, 6076–6085 (2004).

73. X. Zhou, D. K. Halladin, E. R. Rojas, E. F. Koslover, T. K. Lee, K. C. Huang, J. A. Theriot, Mechanical crack propagation drives millisecond daughter cell separation in *Staphylococcus aureus*. *Science* **348**, 574–578 (2015).

74. J. Schindelin, I. Arganda-Carreras, E. Frise, V. Kaynig, M. Longair, T. Pietzsch, S. Preibisch, C. Rueden, S. Saalfeld, B. Schmid, J. Y. Tinevez, D. J. White, V. Hartenstein, K. Eliceiri, P. Tomancak, A. Cardona, Fiji: An open-source platform for biological-image analysis. *Nat. Methods* **9**, 676–682 (2012).

75. J. A. F. Sutton, O. T. Carnell, L. Lafage, J. Gray, J. Biboy, J. F. Gibson, E. J. G. Pollitt, S. C. Tazoll, W. Turnbull, N. H. Hajdamowicz, B. Salamaga, G. R. Pidwill, A. M. Condliffe, S. A. Renshaw, W. Vollmer, S. J. Foster, *Staphylococcus aureus* cell wall structure and dynamics during host-pathogen interaction. *PLOS Pathog.* **17**, e1009468 (2021).

76. E. S. Reynolds, The use of lead citrate at high pH as an electron-opaque stain in electron microscopy. *J. Cell Biol.* **17**, 208–212 (1963).

77. S. F. Altschul, T. L. Madden, A. A. Schäffer, J. Zhang, Z. Zhang, W. Miller, D. J. Lipman, Gapped BLAST and PSI-BLAST: A new generation of protein database search programs. *Nucleic Acids Res.* **25**, 3389–3402 (1997).

78. M. J. Horsburgh, J. L. Aish, I. J. White, L. Shaw, J. K. Lithgow, S. J. Foster, σ^B modulates virulence determinant expression and stress resistance: Characterization of a functional *rsbU* strain derived from *Staphylococcus aureus* 8325-4. *J. Bacteriol.* **184**, 5457–5467 (2002).

79. B. N. Kreiswirth, S. Löfdahl, M. J. Betley, M. O’Reilly, P. M. Schlievert, M. S. Bergdoll, R. P. Novick, The toxic shock syndrome exotoxin structural gene is not detectably transmitted by a prophage. *Nature* **305**, 709–712 (1983).

80. C. Y. Lee, S. L. Buranen, Z. H. Ye, Construction of single-copy integration vectors for *Staphylococcus aureus*. *Gene* **103**, 101–105 (1991).

81. E. L. Cooper, J. García-Lara, S. J. Foster, YsxC, an essential protein in *Staphylococcus aureus* crucial for ribosome assembly/stability. *BMC Microbiol.* **9**, 266 (2009).

82. C. N. Krute, K. L. Krausz, M. A. Markiewicz, J. A. Joyner, S. Pokhrel, P. R. Hall, J. L. Bose, Generation of a stable plasmid for *in vitro* and *in vivo* studies of *Staphylococcus* species. *Appl. Environ. Microbiol.* **82**, 6859–6869 (2016).

83. A. T. F. Liew, T. Theis, S. O. Jensen, J. Garcia-Lara, S. J. Foster, N. Firth, P. J. Lewis, E. J. Harry, A simple plasmid-based system that allows rapid generation of tightly controlled gene expression in *Staphylococcus aureus*. *Microbiology (Reading)* **157**, 666–676 (2011).
